# Exploring mechanisms linked to differentiation and function of dimorphic chloroplasts in the single cell C_4_ species *Bienertia sinuspersici*

**DOI:** 10.1186/1471-2229-14-34

**Published:** 2014-01-21

**Authors:** Josh Rosnow, Pradeep Yerramsetty, James O Berry, Thomas W Okita, Gerald E Edwards

**Affiliations:** 1 School of Biological Sciences, Washington State University, Pullman, WA 99164-4236, USA; 2 Department of Biological Sciences, State University of New York, Buffalo, NY 14260, USA; 3 Institute of Biological Chemistry, Washington State University, Pullman, WA 99164-6340, USA

**Keywords:** Single-cell C_4_ photosynthesis, *Bienertia sinuspersici*, Dimorphic chloroplasts, Chloroplast differentiation

## Abstract

**Background:**

In the model single-cell C_4_ plant *Bienertia sinuspersici*, chloroplast- and nuclear-encoded photosynthetic enzymes, characteristically confined to either bundle sheath or mesophyll cells in Kranz-type C_4_ leaves, all occur together within individual leaf chlorenchyma cells. Intracellular separation of dimorphic chloroplasts and key enzymes within central and peripheral compartments allow for C_4_ carbon fixation analogous to NAD-malic enzyme (NAD-ME) Kranz type species. Several methods were used to investigate dimorphic chloroplast differentiation in *B. sinuspersici*.

**Results:**

Confocal analysis revealed that Rubisco-containing chloroplasts in the central compartment chloroplasts (CCC) contained more photosystem II proteins than the peripheral compartment chloroplasts (PCC) which contain pyruvate,Pi dikinase (PPDK), a pattern analogous to the cell type-specific chloroplasts of many Kranz type NAD-ME species. Transient expression analysis using GFP fusion constructs containing various lengths of a *B. sinuspersici* Rubisco small subunit (*RbcS*) gene and the transit peptide of PPDK revealed that their import was not specific to either chloroplast type. Immunolocalization showed the *rbcL*-specific mRNA binding protein RLSB to be selectively localized to the CCC in *B. sinuspersici*, and to Rubisco-containing BS chloroplasts in the closely related Kranz species *Suaeda taxifolia.* Comparative fluorescence analyses were made using redox-sensitive and insensitive GFP forms, as well comparative staining using the peroxidase indicator 3,3-diaminobenzidine (DAB), which demonstrated differences in stromal redox potential, with the CCC having a more negative potential than the PCC.

**Conclusions:**

Both CCC RLSB localization and the differential chloroplast redox state are suggested to have a role in post-transcriptional *rbcL* expression.

## Background

C_4_ photosynthesis combines two distinct sets of carboxylation reactions that work as a biochemical CO_2_ pump, to increase the efficiency of CO_2_ fixation by ribulose 1,5 bisphosphate carboxylase oxygenase (Rubisco) [1-3]. The first set of C_4_ reactions begins with the initial generation of phosphoenolpyruvate (PEP) by pyruvate, Pi dikinase (PPDK). Then, PEP is used in the carboxylation action of phosphoenolpyruvate carboxylase (PEPC), which mediates the assimilation of atmospheric CO_2_ into C_4_ acids (malate or aspartate). The second set of reactions occurs when the C_4_ acids are transported to an internalized Rubisco-containing cell or compartment, where they are subsequently decarboxylated by either NADP malic enzyme (NADP-ME), NAD malic enzyme (NAD-ME) or PEP carboxykinase (PEPCK), depending on the C_4_ species. C_4_ photosynthesis is typically carried out using a dual-cell system known as Kranz anatomy, where the C_4_ acids are first produced in mesophyll (M) cells, and then transported to the bundle sheath (BS) cells for decarboxylation and re-fixation of CO_2_ by Rubisco. A primary goal of C_4_ photosynthesis research is to understand the process of dimorphic chloroplasts formation and the extent that the chloroplast differentiate from one another in order to support C_4_ biochemistry.

C_4_ differentiation, including the formation of morphologically and functionally dimorphic chloroplasts, occurs during leaf development in both monocot and dicot C_4_ species. The development of full C_4_ capacity progresses from early to mature developmental stages, as structural characteristics and expression patterns for genes encoding various photosynthetic enzymes diverge between the two cell types. Developmental patterns of chloroplast structural and functional differentiation among different C_4_ species show similarities as well as differences [4]. For example, in *Amaranthus hypochondriacus* (NAD-ME type eudicot) and *Zea mays* (NADP-ME type monocot) very early in leaf development Rubisco is initially present in both M and BS cell chloroplasts, in a non-C_4_ pattern, until developmental cues or light signals lead to its restricted accumulation to BS cells alone [5-7]. Complete biochemical and structural differentiation of M and BS cells and the characteristic dimorphic C_4_ chloroplasts is finalized during leaf ontogeny. In addition to differences between the two chloroplast types in Rubisco accumulation, a significant manifestation of this process is differentiation in grana development and the relative levels of components of photosystem I (PS I) versus photosystem II (PS II) in mature leaves [7-10]. The BS chloroplasts of NADP-ME species are deficient in grana stacks due to depleted nuclear encoded PS II components [11], whereas the BS chloroplast of NAD-ME species have more grana development and PS II content compared to M chloroplast [9,12]. While thylakoid differentiation is thought to be regulated mostly by energy requirements (ATP and NADPH) in M and BS cells to support the forms of C_4_, the underlying regulatory mechanism(s) responsible for C_4_-associated differentiation remains a very active area of research [2,13-16].

The control of cell-type specific differentiation in C_4_ species with Kranz anatomy has long been considered to be founded primarily on the opposing transcriptional activation/inactivation of select photosynthetic genes within the nuclei of the distinct M and BS cells. Thus, it was surprising when dimorphic chloroplasts performing full C_4_ photosynthesis were discovered within single chlorenchyma cells of some very unique C_4_ species (see [17]). Over 7,500 C_4_ species are currently known to exist [18], with *Bienertia sinuspersici*, in family Chenopodiaceae, being just one of four known terrestrial species that can perform single-cell C_4_[19,20]. *Bienertia* functions analogous to Kranz C_4_ species, in that its dimorphic chloroplasts work together to concentrate CO_2_ at the site of Rubisco; however, it accomplishes this by their spatial separation between two cytoplasmic domains within individual chlorenchyma cells. Biochemically, *Bienertia* is classified as an NAD-ME type C_4_, with decarboxylation of C_4_ acids in the C_4_ cycle occurring in mitochondria in a cytoplasmic domain known as the central compartment (CC) where the Rubisco-containing chloroplasts are also located [21,22]. The single-cell C_4_ system is thus unique in that there is only one nucleus for the transcription of genes encoding photosynthetic proteins that accumulate specifically within only one cellular compartment and, most notably, within only one of the two compartmentalized chloroplast types. Thus, post-transcriptional processes are, by necessity, required for the selective accumulation of these proteins to develop the dimorphic, compartmentalized chloroplasts which are required for C_4_ function in these plants.

Rubisco is a hetero-octomer, composed of an equal number of large subunits (rbcL) transcribed and translated in the chloroplast and small subunits (RbcS) that are transcribed in the nucleus, translated in the cytoplasm, and imported into the chloroplast [23]. As observed in some Kranz species, in the single-cell C_4_ species *Suaeda aralocaspica* and *Bienertia cycloptera*, Rubisco is initially present in both chloroplast types early in development [22,24]. There have been numerous studies exploring Rubisco assembly, which show that rbcL and RbcS subunits accumulate in equal amounts within the chloroplast stroma, and the loss of either peptide will cause a decrease in the other subunit peptide [25]. Recently, an RNA binding protein known as RLSB has been shown to correlate with BS specific localization of Rubisco in both monocot and dicot C_4_ plant species. RLSB is hypothesized to be necessary for *rbcL* mRNA maturation and translation in both C_3_ and C_4_ plants [26]. There are various ways that selective accumulation of Rubisco to the chloroplasts in the CC of *Bienertia* could be controlled; e.g. selective chloroplast targeting of nuclear encoded proteins such as the RbcS itself, a Rubisco-associated chaperonin such as Raf1 [27] or RLSB. Other mechanisms could include the selective transcription or translation of *rbcL* within the CCC, regulated assembly, selective degradation of necessary assembly chaperones, or some combination of these mechanisms.

*In situ* immunolocalization and western blots using isolated chloroplasts have demonstrated selective accumulation of Rubisco within the CCC of mature leaves of *Bienertia*[21,22]. These methods have also shown the selective compartmentalization of PPDK to the other chloroplast type within the peripheral compartment (PC). In addition to these selectively targeted proteins between these two chloroplasts, there are other nuclear-encoded proteins with a biochemical role in both chloroplast types that would need to be dual targeted. This group includes the enzyme pyrophosphatase [which is required in starch synthesis in the CCC and in the generation of phosphoenolpyruvate (PEP) from pyruvate in the PCC], and enzymes of the reductive phase of the C_3_ cycle (phosphoglycerate kinase and glyceraldehyde-P dehydrogenase) [21]. The goal of the current study was to investigate the dimorphic chloroplasts of *Bienertia*, with a central focus on mechanisms that may be responsible for the selective accumulation of Rubisco and PPDK, two enzymes that are specific to different chloroplast types located in separated domains. Specifically, we have investigated the possible selective targeting of RbcS to the CCC and PPDK to the PCC, through the use of constructs containing various lengths of the *RbcS* transcript fused to GFP, as well as the transit peptide of PPDK, by use of biolistic and protoplast transformation of *Bienertia* chlorenchyma cells. Cellular localization of the RLSB protein was determined as another possible control mechanism of rbcL synthesis. To assess the redox status as a potential control mechanism for each chloroplast type, fluorescence from a redox sensitive GFP (roGFP2) was measured, and an *in vivo* peroxidase activity and hydrogen peroxide (H_2_O_2_) stain was quantified. We discuss evidence that overlapping regulatory processes could act as determinants in the formation and function of dimorphic chloroplasts in this single cell C_4_ system.

## Results

### GFP expression analysis

*Summary of GFP Constructs*. The set of GFP fusion constructs were made by placing *Bienertia* c-DNA (except the AGPase construct [28]) on the N-terminus of the GFP protein (except the 3′UTR of RbcS). A list of the constructs used and results are summarized in Additional file 1: Table S1. Briefly, puc18 spGFP is a positive control lacking a targeting signal. PPDK constructs contained the first 180 or 273 protein-encoding nucleotides (PPDK-180 spGFP and PPDK 273 spGFP) while PPDK-CDS spGFP has the entire PPDK coding sequence (CDS). RbcS constructs contained the first 252 or 273 protein-encoding nucleotides (RbcS-252 spGFP and RbcS-273 spGFP) of the most abundant of three *Bienertia RbcS* transcripts [29] (R. Sharpe, unpublished), while RbcS-CDS spGFP has the entire RbcS CDS. Additional *RbcS* UTR sequence was added in various combinations to the RbcS constructs, with the RbcS-FL spGFP construct containing the entire *Bienertia RbcS* transcript. Alternative RbcS-FL spGFP constructs included using the super ubiquitin promoter (pSU) with an intron (pSU RbcS-FL spGFP) and re-placing spGFP protein with the roGFP2 protein (RbcS-FL roGFP2). A construct containing the entire CDS of the *Bienertia* RLSB c-DNA was fused in frame to the N-terminus to the GFP protein (RLSB CDS spGFP).

*Biolistic Transformation.* Initially biolistic transformation was used to test for expression and plastid targeting of GFP constructs in *Bienertia* leaves compared with onion epidermal cells (see results with biolistics in Additional files 2, 3, 4, 5). In onion, various constructs were expressed in epidermal cells, and in many cases import into pro-plastids was observed. In *Bienertia* most constructs were expressed in chlorenchyma cells; but, only rarely was import into chloroplasts observed (i.e. see evidence for import into both PCC and CCC with construct PPDK-180 spGFP and RbcS-273 spGFP, Additional files 3 and 4: Figure S2 and S3). Due to the limited occurrence of import in *Bienertia* with biolistics, to further evaluate expression and targeting using these constructs, protoplast transformation was used.

**Figure 1:**
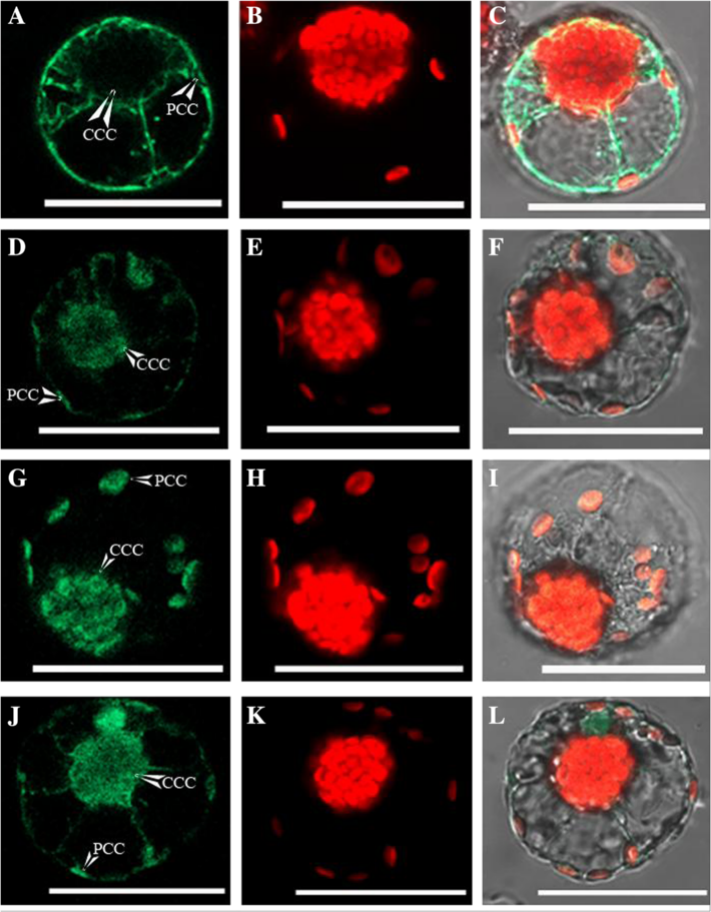
**Confocal images of GFP expression in *Bienertia *protoplasts. A** - **C** is spGFP (positive control), **D** – **F** is PPDK180-spGFP, **G** – **I** is RbcS-273 spGFP, **J** – **L** is pSU RbcS-FL spGFP. Images **A**, **D**, **G**, and **J** are GFP emission. Images **B**, **E**, **H**, and **K**, are chlorophyll autofluorescence emission. Images **C**, **F**, **I**, and **L** are the merged images of GFP and chlorophyll autofluorescence emission. CCC = central compartment chloroplast, PCC = peripheral compartment chloroplast. Scale Bar = 50 μm.

*Plastid targeting of GFP constructs using protoplast transformation.* A summary of the results with transient expression of constructs in *Bienertia* chlorenchyma protoplasts shows a consistent high co-occurrence of import with that which occurs in onion by biolistic treatment (Additional file 1: Table S1). The puc18-spGFP positive control showed GFP expression throughout the cytoplasm in *Bienertia*, with no GFP fluorescence observed within any of the chloroplasts (Figure 1, A-C), as also observed with the biolistic method with onion and *Bienertia*. The PPDK180-spGFP construct showed significant levels of GFP expression in both the PCC and CCC (Figure 1, D-F). Also, the RbcS273-spGFP construct produced clearly observable GFP expression in both the PCC and the CCC (Figure 1, G-I). The pSU-RbcS full length-spGFP construct showed GFP expression in both the PCC and CCC, with some GFP accumulation observed within the nucleus as well (Figure 1, J-L) (to some extent GFP translocation occurs into the nucleus [30]). Results for the RLSB-spGFP construct in *Bienertia* protoplasts were inconclusive, due to very low levels of GFP expression. Quantitative measurements are not shown for constructs that had similar GFP fluorescence from the PCC and CCC.

**Figure 2:**
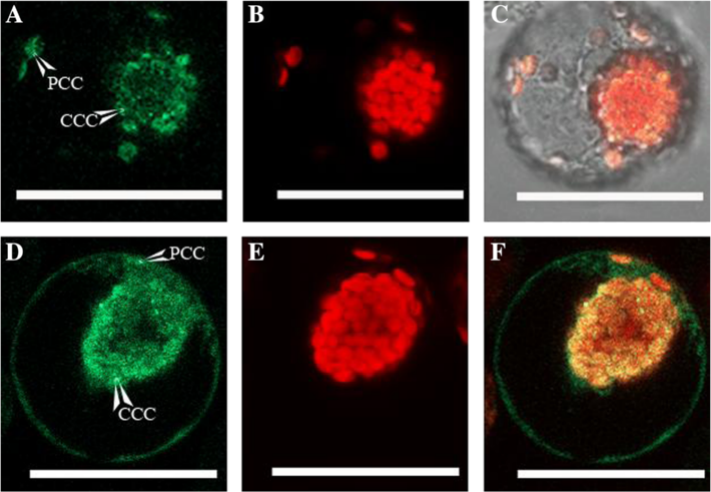
**Confocal images of GFP expression in *Bienertia *protoplasts from the construct RbcS-FL spGFP (A-C) and the construct RbcS-FL roGFP2 (D-F).** The roGFP2 protein is redox sensitive, while the spGFP protein is redox insensitive. Cells were scanned for a focal plane closest to the slide surface to maximize GFP fluorescence from each chloroplast type. Images **A** and **D** are GFP emission. Images **B** and **E** are chlorophyll autofluorescence. Images **C** and **F** are the merged images of GFP and chlorophyll autofluorescence emission. CCC = central compartment chloroplast, PCC = peripheral compartment chloroplast. Scale Bar = 50 μm.

Because of the high sensitivity for the detection of GFP expression and targeting, as well as the lack of selectivity for import into either chloroplast type, the protoplast transformation method was used to determine the relative redox states of the dimorphic chloroplasts of *Bienertia*. For this purpose, protoplasts were transformed using the RbcS-FL construct linked with spGFP (redox insensitive) or roGFP2 (redox sensitive) reporter proteins (Figure 2). Representative data of confocal quantification of GFP fluorescence from the dimorphic chloroplasts of *Bienertia* protoplasts is shown in Additional file 6: Figure S5. Both constructs, which only differ in the redox sensitivity of their attached GFP protein, correctly target GFP to both chloroplast types (Figure 2, A and C). Comparatively analyzing GFP fluorescence between the two-chloroplast types indicted that fluorescence intensity from the spGFP protein was similar in both, while the roGFP2 protein produced a higher intensity of GFP fluorescence within the CCC than within the PCC. A summary of the confocal microscopy quantification of GFP fluorescence from transformed *Bienertia* protoplasts using Lambda mode, which quantifies fluoresence intensity from multiple locations, is presented in Table 1. The results show that the average CCC/PCC ratio of GFP fluorescence when using the redox insensitive spGFP protein was 1.028 ± .024, while the average ratio of GFP fluorescence between the two chloroplast types when using the redox-sensitive roGFP2 protein was 1.439 ± .005. The fluorescence of the roGFP2 protein, when excited with 488 nm, is known to decrease as the redox potential of its environment is decreased [31]. Attempts to determine an exact mid-point potential of the two chloroplast types were made, by making ratiometry measurements of fluorescence. However, the fragile nature of the protoplasts were not suitable for such measurments, due to the requirement for calibration of the system by additional washings of the protoplasts with reducing and oxidizing solutions.

**Table 1:** **The average CCC/PCC ratio of GFP fluorescence from the dimorphic chloroplast of transformed *Bienertia *** **protoplasts, using the constructs RbcS-FL spGFP and RbcS-FL roGFP2, where the roGFP2 protein is redox sensitive while the spGFP protein is redox insensitive**

**Construct**	**# of quantified cells**	**Average CCC/PCC ratio of fluorescence**
RbcS-FL spGFP	31	1.028 +/− 0.024*
RbcS-FL roGFP2	28	1.439 +/− 0.005*

Lambda mode on the Zeiss 510 confocal microscope was used to measure the intensity of GFP fluorescence. Fluorescence intensity values were obtained using an excitation wavelength of 488 nm, and detection of emission at a wavelength of 513 nm. (Additional file 6: Figure S5 shows a picture of the analysis). The ratio of GFP fluorescence intensity between the two chloroplast types was calculated by dividing the intensity of one central compartment chloroplast by the intensity of one peripheral compartment chloroplast. The ratio was averaged across the number of quantified cells. The results presented are from 7 biological replicates. The standard error from all measurments is shown. *Difference between the two constructs are statistically significant at p < 0.05, using a independent T-test.

### Immunolocalization

**Figure 3:**
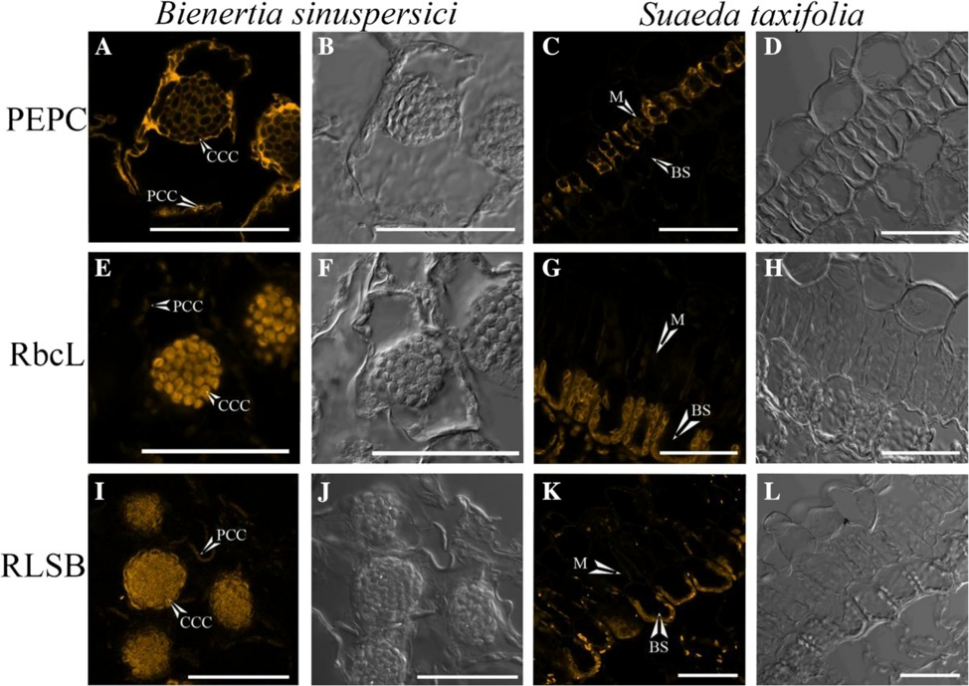
**Immunolocalization of C**_**4 **_**proteins in single cell type *Bienertia *and Kranz type *Suaeda taxifolia*.** Confocal microscopy detection of the Alexa fluor 546 tagged secondary antibody, reacting to the primary antibodies indicated, showing the location of phospho*enol*pyruvate carboxylase (PEPC, **A-D**), rubisco large subunit (rbcL, **E-H**), and rubisco large subunit mRNA binding protein (RLSB, I-L) in *Bienertia***(A, B, E, F, I, and J)** and *Suaeda taxifolia***(C, D, G, H, K, and L)**. Images **A**, **C**, **E**, **G**, **I**, and **K** are Alexa Fluro 546 detection. Images **B**, **D**, **F**, **H**, **J**, and **L** are bright field view of sections. CCC = central compartment chloroplast, PCC = peripheral compartment chloroplast, M = Mesophyll Cell, BS = Bundle Sheath Cell. Scale bar = 50 μm.

Immunolocalization was used to determine the cellular localization of the C_4_-associated proteins PEPC, rbcL, and the regulatory RLSB within *Bienertia* leaf chloroenchyma cells, and, for comparison, in the closely related Kranz-type species *S. taxifolia* (Figure 3). Fluorescence from an Alexa fluor 546-tagged secondary antibody, reacting with the primary antisera was detected using confocal microscopy. The analysis demonstrated that PEPC, as expected, was distributed throughout the cytoplasm in *Bienertia* chloroenchyma cells, and was selectively localized in M cells of *S. taxifolia* leaves (Figure 3, A-D). rbcL showed selective localization within *Bienertia* chloroplasts, with very high levels of intensity occurring only within the CCC. In comparison, the rbcL protein was highly specific to the BS chloroplasts of *S. taxifolia* (Figure 3, E-H), in the characteristic pattern for Kranz leaves. Most significantly, the regulatory RLSB protein, like the rbcL protein it is proposed to regulate, was selectively localized only within the CCC of *Bienertia* chlorenchyma cells. In agreement with previous findings [26], RLSB accumulation was observed to be highly specific to the BS chloroplasts in Kranz leaves of *S. taxifolia* (Figure 3, I-L).

It should be noted that within the *Bienertia* chlorenchyma cells, both rbcL and RLSB proteins showed very slight levels of detection within the PCC, possibly due to very low levels of accumulation, or possibly background levels of reaction within these chloroplasts. Taken together, these findings clearly demonstrate strong specific localization of RLSB to the Rubisco-containing CCC of *Bienertia* chlorenchymeca cells, and the BS cell chloroplasts of a closely related but structurally distinct C_4_ species.

### DAB staining to test for relative redox state of CCC and PCC in *bienertia*

The use of scanning electron microscopy (SEM) backscattering to quantify the precipitation of DAB particles in *Bienertia* tissues proved to be more reproducibly accurate than light microscope or transmission electron microscopy (TEM) images (images not shown). This can be attributed to the limited handling requirements for SEM sample preparation, as well as reducing variability due to sectioning and staining protocols required for light microscope and TEM. The backscattering mode on the SEM produces images that correspond tightly to the density of electron particles present. The more electrons present in an area the brighter the area will be in the image. In contrast, where there are few electrons (i.e., only water present, as in the vacuole space), electron density is low, and the area appears black. Various staining times, and light intensities were tested, and conditions that maintained cell morphology and optimized visualization/quantification of staining levels were used for analysis.

**Figure 4:**
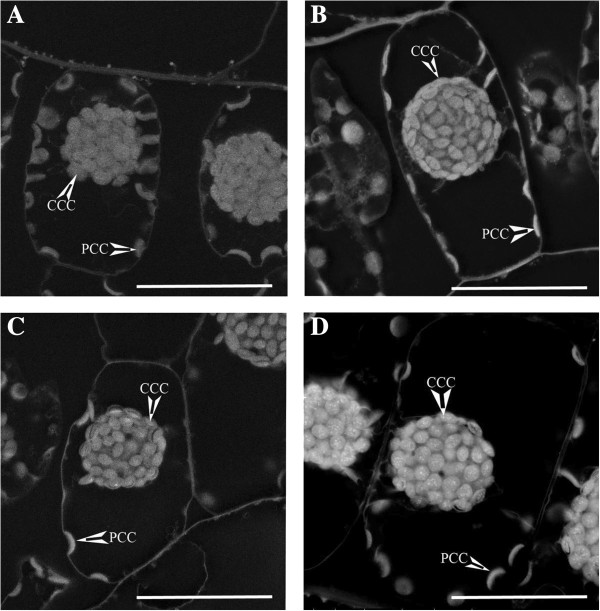
**Representative images from SEM backscattering detection of DAB stained *Bienertia *chlorenchyma cells (For quantification of results see Table **2**).** Image **A** is the control for H_2_O_2_ detection (no DAB was included). Image **B** is the stain for H_2_O_2_, where DAB was allowed to react with *in vivo* produced H_2_O_2_. Image **C** is the control for peroxidase activity (no H_2_O_2_ was added). Image **D** is the stain for peroxidase activity, where H_2_O_2_ is supplied in excess. CCC = central compartment chloroplast, PCC = peripheral compartment chloroplast. Scale Bar = 50 μm.

To probe for relative steady state levels of H_2_O_2_ generation in the dimorphic chloroplasts in the light, *Bienertia* leaves were excised and incubated under 100 PPFD (this low light intensity was used to avoid damaging the cells) in the presence of DAB (H_2_O_2_ stain) versus in the absence of DAB (control). Quantification of electron densities revealed that in the absence of DAB (negative control), the CCC was brighter in comparison to the PCC, with an average density ratio of 1.658 ± 0.128 (Figure 4-A, Table 2). When DAB was included in the staining reactions to detect H_2_O_2_ production, the PCC became brighter relative to the CCC, with the average density ratio dropping to 1.265 ± 0.097 (Figure 4-B, Table 2). Quantification and comparison of the CCC to PCC integrated density ratios showed that the density ratio of particles decreased when staining for H_2_O_2_, suggesting that more DAB was precipitated in the PCC than in the CCC. This is indicative of a higher level of H_2_O_2_ in the PCC relative to the CCC, which interacts with and precipitates DAB under these steady state conditions.

**Table 2:** **SEM backscattering quantification of *Bienertia *** **DAB staining, using Image J analysis to quantify the Integrated Density of the two chloroplast types**

**Stain**	**Total average CCC/PCC density ratio**
Control stain	1.658 +/− 0.128*
H_2_O_2_ stain	1.265 +/− 0.097*
Peroxidase control stain	1.196 +/− 0.058*
Peroxidase stain	1.965 +/− 0.239*

The density ratio was calculated by dividing the average integrated density of 5–7 central compartment chloroplasts (CCC) by the average integrated density of an equal number of peripheral chloroplast (PCC) for each cell. The total average CCC/PCC density ratio was obtained by averaging the density ratio from two biological replicates (3 cells each) and two technical replicates. The standard error is shown for all 12 measurements. ^*^Difference between the control and stain are statistically significant at p < 0.05, using a independent T-test.

To test for difference in the ability of the two chloroplasts to scavenge and reduce H_2_O_2_, a peroxidase activity stain was performed by adding H_2_O_2_ to the DAB staining solution. Excised leaves were placed in the DAB staining solution in the absence of H_2_O_2_ (peroxidase control where *in vivo* H_2_0_2_ is limiting), or in the DAB staining solution with 7 mM H_2_O_2_ (peroxidase stain) added. Analysis of the images indicated the two chloroplast types had a similar appearance with the control (H_2_O_2_ minus) peroxidase stain. In this control, the CCC was only slightly brighter than the PCC, with an average density ratio of 1.196 ± 0.058 (Figure 4-C, Table 2). When H_2_O_2_ was included in the stain to detect peroxidase activity, the CCC became significantly brighter than the PCC, so that the average density ratio rose to 1.965 ± 0.239 (Figure 4-D, Table 2). Upon closer observation of the peroxidase stain images, the CCC had more visible precipitation dots in comparison to PCC or the control images. Quantification and comparison of the CCC to PCC integrated density ratios showed that the density ratio increased significantly in this peroxidase activity stain. The results indicate that there is more peroxidase activity in the CCC relative to the PCC, under the steady state conditions of this assay.

### Estimation of PSII Content between the two chloroplast types

**Figure 5:**
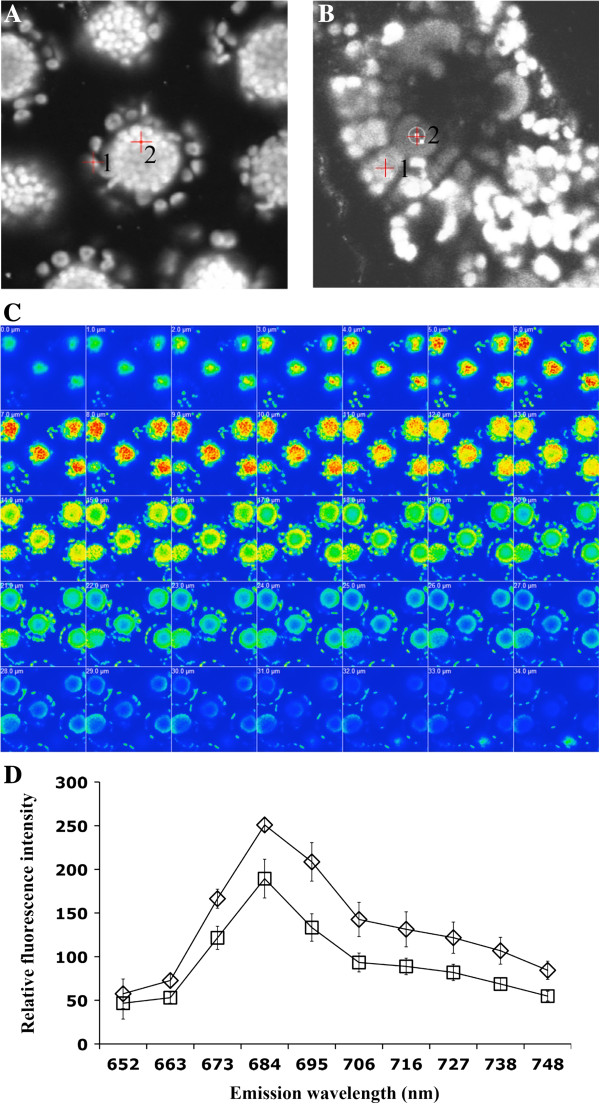
**Estimation of PSII content in *Bienertia *chlorenchyma cells, using confocal microscopy.** Cells were excited with 633 nm of light, and the brighter areas in images **A**, **B**, and **C** have greater fluorescence intensities, corresponding to more PSII content. **A)** Lambda mode image showing fluorescence from the two chloroplast types of *Bienertia*. Cursor 1 was placed on one peripheral chloroplast, while cursor two was placed on one central compartment chloroplast. **B)** Lambda mode image showing fluorescence from the two chloroplast types of *Zea mays*. Cursor 1 was placed on the mesophyll chloroplasts, while cursor two was placed on the bundle sheath. **C)** Heat map of a z-stack (serial images) series of a *Bienertia* protoplast at 1 μm slices, intensity represents the emission at 684 nm using 633 nm excitation. **D)** Quantification of the emission spectra of the two types of *Bienertia* chloroplasts; squares are peripheral compartment chloroplasts, and diamonds are central compartment chloroplasts. Error bars represent the standard deviation for 8 different protoplasts.

Quantification of chloroplast fluorescence emitted from PSII has been shown to be a rapid and efficient method for assessing differences in the PSII content in different chloroplast types of C_4_ species [32]. The application of this analysis to *Bienertia* protoplasts demonstrated that the CCC had a greater intensity of fluorescence intensity in comparison to the PCC (Figure 5-A), indicative of a higher PSII content in the CCC. As a comparative control, this same analysis was performed using a fresh cross-section of a *Zea mays* leaf, a Kranz type C_4_ species that is known to have low PSII content in BS chloroplasts (Figure 5-B). As expected, in this species the PSII-related fluorescence was greater from M (PSII containing) than BS (PSII reduced) chloroplasts.

A heat map z-scan from a whole intact *Bienertia* protoplasts is shown in Figure 5-C, where the intensity of the heat signal (with red as the highest) corresponds to fluorescence at 684 nm. Within each protoplast frame, it can be seen that all of the detectable fluorescence emitted from the CCC has a much higher intensity than that emitted from the PCC. When averaged across 8 protoplasts, there is 1.3 times higher PSII associated fluorescence emitted from CCC compared to PCC (Figure 5-D).

## Discussion

### Photosystem II content in central and peripheral chloroplasts and its relevance to C_4_ biochemistry in *Bienertia*

Comparative confocal quantification is an established technique for determining the relative PS II content in M and BS cells of C_4_ species [32,33]. Applying this methodology to the dimorphic chloroplasts of *Bienertia* showed that the density of PSII is substantially higher in the CCC, which suggests higher capacity for linear electron flow than in the PCC. These relative amounts are comparable to prior quantitative analysis of dimorphic chloroplast in the single cell C_4_*Bienertia cycloptera*, which were shown to have a CCC granal index (the percentage of thylakoid membranes which are appressed) that is 1.5 times higher than the PCC [34]. This is typical for Kranz type species with NAD-ME biochemistry, with some species having up to 2-fold higher chloroplast granality in BS compared to M cells [9,33]. These results are consistent with the general assessment that there is an enrichment of PSII in BS chloroplasts relative to M chloroplasts in NAD-ME type species.

Chloroplast ultrastructural differentiation observed at the mature cell stage of *Bienertia* could be the result of a progressive establishment of C_4_ biochemistry during leaf ontology, since early in development there is little difference in the thylakoid membranes of the two chloroplasts [22]. This is true for other C_4_ species, where differences in the granal index of different chloroplast types are observed only later in development [35]. In mature leaves of Kranz-type NAD-ME species the only energy requirements in the C_4_ cycle is 2 ATP per CO_2_ delivered to the BS cells. For single cell C_4_ species such as *Bienertia*, this requirement for ATP occurs in the PCC to support the conversion of pyruvate to PEP. Thus there is a greater energy requirement in the CCC (as in the BS chloroplasts of NAD-ME Kranz species), where NADPH needs to be generated by PSII dependent electron transport to support the C_3_ cycle [4]. As C_4_ leaf development progresses, aspects of C_4_ biochemistry between the PCC and the CCC diverge, with the demand for NADPH decreasing in the PCC and increasing in the CCC. In mature leaf cells, reduced NADPH utilization in the PCC would likely cause an acceptor limitation for electrons derived from PSII activity, resulting in inactivation of PSII by generation of singlet oxygen, and a corresponding increase in cyclic electron flow for ATP generation by PSI [36,37]. This type of biochemical regulation of PSII content has also been demonstrated in transgenic rice (a C_3_ plant) by expressing *Zea mays* NADP-ME in the chloroplasts that resulted in reduced development of grana and PSII activity. This was suggested to be caused by increased uptake and decarboxylation of malate by NADP-ME in the chloroplasts which generates reductive power leading to reduced need for photosynthetic production of NADPH via PSII [38]. These studies, together with findings presented here, support a model in which the basic requirement for energy production, in itself, could be a determinant for the regulation of PS I and PS II development. Such dynamic regulation could ultimately balance the production/accumulation of the two photosystems according to the needs of each chloroplast type.

### Mechanisms for selective chloroplast accumulation of nuclear encoded proteins

The unique single-cell C_4_ system of *Bienertia* differs significantly from the two-cell system found in the more typical Kranz species. By necessity this system is primarily regulated by post-transcriptional control processes that achieve selective accumulation of enzymes in the dimorphic chloroplasts. Several hypotheses have been recently proposed for differential protein localization in this system, including selective chloroplast protein import, mRNA trafficking to specific domains for translation, and/or the selective proteolysis after protein import [39].

Analysis of the various RbcS and PPDK GFP fusion constructs were used in this study to test for selective chloroplast import. The results showed that similar levels of GFP uptake occurred in both chloroplasts types in *Bienertia* chlorenchyma cells (Figure 1 and Additional files 3 and 4). A similar observation was made with RbcS in an earlier study, where an undefined RbcS sequence was shown to direct GFP import into both chloroplast types within *Bienertia* protoplasts [40]. Taken together, the *in vivo* biolistic and protoplast analysis of this current study clearly demonstrate that the transit peptide of PPDK or various lengths of the most abundant RbcS proteins, in themselves, were capable of directing at least some level of GFP fusion uptake, depending on the plant system used. However, the protein expressed from these constructs showed no evidence of selectivity for either *Bienertia* chloroplast type. The lack of selectivity in GFP targeting could also suggest that additional sequence information that was not included in the constructs might be needed for selective chloroplast targeting. This could include additional mRNA sequences (introns or more UTR), alternative gene family members in the case of *RbcS*, or differential placement of the GFP CDS. There could also be experimental effects, such as a difference in recognition and import of native peptides versus the peptide fragments, or regulation in intact leaves that becomes disrupted in protoplasts. For example, selective import/targeting could be disrupted during protoplast isolation and overnight incubation. However, observations by light microscopy showed the two cytoplasmic domains were maintained (which is dependent on integrity of the cytoskeleton [41]).

One potential mechanism for selective targeting of proteins within *Bienertia* chloroenchyma cells is the trafficking of mRNA facilitated by mRNA-binding protein (RBP) complexes. While cytoplasmic mRNAs typically associate with RBPs to control their stability or initiate and maintain translation on free ribosomes [42], there are some RBPs that can move selected mRNAs along the cytoskeleton to destinations within specific subcellular domains. As an example, some RBPs in plant cells can mediate trafficking to defined regions of the endoplasmic reticulum (ER). The most studied example is the OsTudor-staphylococcal nuclease (SN) RBP that facilitates the movement of prolamine mRNA along actin filaments to the cortical ER in rice endosperm [43]. However, to date there have been no reports of RBPs responsible for localizing nuclear-encoded photosynthetic transcripts, such as *RbcS* or C_3_ cycle enzymes, to specific domains of a photosynthetic cell (i.e. closer to the chloroplast for more efficient chloroplast targeting).

At least some regions necessary for *RbcS* regulation occur in the mRNA UTRs. In rice leaves, it was shown that for mRNA turnover to occur, there needs to be both the 5′ and 3′ UTR of *RbcS* present [44]. Similar results have been found in the C_4_ plants *Zea mays* and *Cleome gynandra*, where the *RbcS* UTRs or portions upstream of the gene (1.0 kb of *ZmRbcs & 3*.8 kb of *CgRbcS* gene region) were sufficient to confer BS specific RbcS or β-glucuronidase accumulation [45-47]. In themselves, the 5′ and 3′ UTRs of a heterologous *RbcS* mRNA from *Amaranth,* constitutively expressed in *F. bidentis* leaves, were sufficient to confer partial BS cell specificity of a β-glucuronidase fusion [45]. Taken together, these results imply that in the dual-cell Kranz system, interaction of UTRs with specific RBP(s) might enhance translation/stability in the BS cells, or perhaps decrease stability/translation in M cells. Further experimentation will be needed to address whether *RbcS* regulation in the cytoplasm is analogous to *rbcL* regulation in the chloroplasts, i.e. whether an RBP such as the chloroplastic RLSB [26] might be capable of interacting with and mediating RbcS transcript stability/translation in the cytoplasm. While this seems like a plausible mechanism, no *Rbc*S mRNA interacting proteins of any type have been identified. In this study, the addition of the pSU intron upstream of the *RbcS* 5′UTR, along with the pSU plant promoter did not change the results of non-selective GFP fluorescence from the dimorphic chloroplasts (Figure 1). Thus, even when using a plant promoter with upstream RNA sequences and an intron, there was no evidence for transcript-mediated selectivity for single-cell type compartmentalization *in vivo.*

In agreement with our findings, recent analyses on the translocon of the outer envelope membrane of chloroplasts (TOC) of *Bienertia* using various chloroplast targeted TOC-GFP constructs and *Bs*TOC159, *Bs*TOC132, and *Bs*TOC34 antibodies showed that both chloroplast types import and accumulate nuclear encoded TOC proteins non-selectively similarly to other plants [40,48]. The current understanding of stromal and TOC protein import suggests that the main role of cytosolic targeting factors/chaperones in chloroplast localization is to increase targeting efficiency and uptake rate by maintaining peptide recognition competency [49,50]. The only factor that has been determined to be necessary for the proper sorting to the stroma is a transit peptide, which interacts with the stromal HSP70 chaperone and the outer envelope membrane [49,51,52]. One interesting aspect of chloroplast protein import, is the regulation that has been shown to occur by the redox regulated Tic62 protein, which changes its location based on the stroma NADP^+^/NADPH ratio [53,54]. There have been no previous reports for Kranz C_4_ species that regulation occurs at the chloroplast import level.

In summary, the findings presented here, together with those of previous studies, have revealed no evidence for the chloroplast import machinery, selective mRNA trafficking, or compartment-selective activation/translation of nuclear-encoded photosynthetic mRNAs, having a direct role in selective protein accumulation for the dimorphic chloroplasts in *Bienertia*. Therefore, C_4_ regulation in the single-cell system must involve additional regulatory processes, acting either independently or in synergy with one or more of the mechanisms described above.

### Rubisco synthesis, assembly, and relevance to selective localization in *Bienertia* dimorphic chloroplasts

Post-transcriptional regulation (translation and stability) is the primary level at which plastid-encoded protein such as rbcL are regulated. Similar to nuclear-encoded transcripts, the UTRs of plastid-encoded mRNAs play a key role in their regulation. Many studies have demonstrated that such regulation of plastidic RNA metabolism occurs through interactions with groups of nuclear-encoded chloroplast RBPs. These proteins fall into several classes, with molecular masses ranging from 38–60 kDa [55-58]. Some of these have been shown to assemble on the 5′UTR of *psbA, psbC*, and *rbcL*, and other plastidic transcripts [56,57]. For many of these proteins, their production, binding, or activity are often determined by light- or redox potential, such that binding to the 5′ UTR under activation conditions might enhance the mRNA′s translation, processing, or stability [58,59].

In the case of *rbcL* mRNA, it has been shown that the rbcL protein itself has an N-terminal RNA binding domain that could have a role in mediating its own translational arrest by binding its own 5′ UTR [60]. To date, MRL1 and RSLB are the only two nuclear-encoded proteins to be directly implicated in the regulation of *rbcL* mRNA metabolism through interaction with its 5′ regions [26,61]. The number of chloroplast regulatory factors required for the correct assembly of Rubisco continues to increase, with RLSB and RAF 1 & 2 being the most recent additions [26,27]. Both BSD2 and RAF1 have been identified as rbcL assembly factors that are necessary for the proper assembly of Rubisco within the BS chloroplasts of C_4_ plants [27,62]. In *Zea mays*, RAF1 is specific to BS chloroplasts, whereas BSD2 is localized in both M and BS chloroplasts*,* indicting different regulatory roles for these functionally-related proteins. Thus, the selective accumulation of the Rubisco holoenzyme in the CCC of *Bienertia* could involve a compartmentally-selective increase in either *rbcL* mRNA binding proteins or rbcL assembly chaperones. These mechanisms are not exclusive, and both could work together to achieve differential Rubisco accumulation in mature *Bienertia* leaf cells.

The results show that RLSB is highly specific to the CCC in *Bienertia* cells, co-localizing with rbcL within this same chloroplast type, which is clearly distinct from the cytosolic localization of PEPC (Figure 3). The cellular localization shown for rbcL and PEPC is the same as previously reported for *Bienertia*[22,34]. The co-localization of RLSB to the Rubisco-containing chloroplasts provides evidence for a regulatory role in the selective compartmentalized synthesis of rbcL in this single-cell system. As a comparison, RLSB was also found to be specifically localize to the rbcL-containing BS chloroplasts of the related Kranz type *S. taxifolia*, in agreement with its BS-specific localization and proposed regulatory role in the Kranz C_4_ species *Flaveria bidentis*, *Zea mays* and *Setaria viridis*[26]. Selective localization of RLSB to the CCC in *Bienertia* suggests that, as in *Zea mays*, this protein may function in the post-transcriptional regulation of rbcL synthesis, binding to *rbcL* mRNA to enhance its translation/stability specifically within these chloroplasts.

As with other C_4_ proteins in this unique single-cell system, mechanisms responsible for the selective accumulation of RLSB within the CCC are as yet unknown. Possibilities include selective import to the CCC, or possibly selective degradation of RLSB in the PCC if it was unable to bind to its target *rbc*L mRNA. Attempts to determine if RLSB could be selectively targeted to the CCC when fused to GFP were inconclusive using *Bienertia* protoplast transformation, due to the low level of GFP expression from this construct. Some *Chlamydomonas reinhardtii* proteins responsible for rbcL translation have been shown to dissociate from the *rbcL* 5′ UTR upon increasing concentrations of oxidized glutathione; these are subsequently replaced through binding of the rbcL protein to its own mRNA [60]. A similar processes was suggested to occur in the higher plant *Populus deltoides*[63]. If such self-regulation were to occur in the *Bienertia* PCC, then it might be expected that a basal level of rbcL protein would always be present, as a way to prevent its own translation. In fact, low levels of rbcL were detected in the PCC by immunolocalization, although it is uncertain whether this is free large subunit, the Rubisco holoenzyme, or possibly low levels of background due to non-specific interactions of the primary antibody (Figure 3, N. Koteyeva and E. Voznesenskaya, unpublished observations).

In *Nicotiana tabacum* and *Zea mays*, it was previously shown that in the absence of RbcS, rbcL is subject to translational repression, through an interaction of unassembled rbcL with its encoding *rbcL* transcript [control by epistasy (CES)], a posttranslational regulation mechanism [25,64]. If CES exists in C_4_ plants, by transcriptionally limiting the availability of RbcS for Rubisco assembly (perhaps in *Bienertia* through selective chloroplast targeting), than translation of rbcL would be repressed, thereby impeding Rubisco assembly. However, GFP fusion constructs possessing different lengths of an abundant *RbcS* transcript did not support the selective transport aspect of this hypothesis. Fusing GFP to the C-terminal of the RbcS transit peptide, the RbcS CDS, or even the entire *RbcS* transcript (with the *RbcS* 3′UTR sequence being attached to the C-terminus of GFP), indicated that the RbcS protein can be imported into both chloroplast types, with no import selectivity identified (Figure 1). These results are in agreement with a recent analysis in *Zea mays*, where ectopic expression of RbcS in the M cells, alone or in combination with the expression of a nuclear-encoded version of an rbcL peptide targeted to chloroplasts, did not lead to significant accumulation of Rubisco in M cells [64]. Taken together these results indicate that selective RbcS targeting, and an associated inability to assemble Rubisco within one chloroplast type, are not in themselves responsible for the selective compartmentalization of Rubisco in *Bienertia* leaves.

Changes to the stromal redox state within the chloroplasts have been shown to affect *rbcL* translation as well as Rubisco enzymatic function [65,66]. Synthesis of rbcL has also been found to correlate with changes in ROS production, detoxification, and the ratio of GSH/GSSG within the chloroplasts [67,68]. The intact Rubisco enzyme itself is known to be susceptible to H_2_O_2_. Low concentrations lead to enzyme inactivation; increasing concentrations can cause unfolding and the loss of secondary and tertiary structure, ultimately leading to increased protease susceptibility [65,69]. Thus, another hypothesis for differential Rubisco accumulation in the two chloroplasts types is based on stromal redox conditions. This would occur if the CCC had a higher GSH/GSSG ratio compared to the PCC, and the CCC had a lower H_2_O_2_ concentration and greater detoxification capabilities. A difference in the redox state of the stroma is supported by the observation that roGFP2, which most directly reflects the ratio of GSH/GSSG, has altered fluorescence in the two *Bienertia* chloroplast types. Results with DAB staining also suggest that the PCC has a higher *in vivo* H_2_O_2_ concentration, with the CCC having more peroxidase activity. The level of H_2_O_2_ in the chloroplast and the redox state of GSH/GSSG depends on a number of factors including the capacity of the chloroplast for linear electron flow, the demand of NADPH for carbon assimilation, the extent of the Mehler reaction (electron transfer from PSI to O_2_ leading to H_2_0_2_ synthesis), and the capacity to scavenge H_2_O_2_ using antioxidants ascorbate and glutathione.

**Figure 6:**
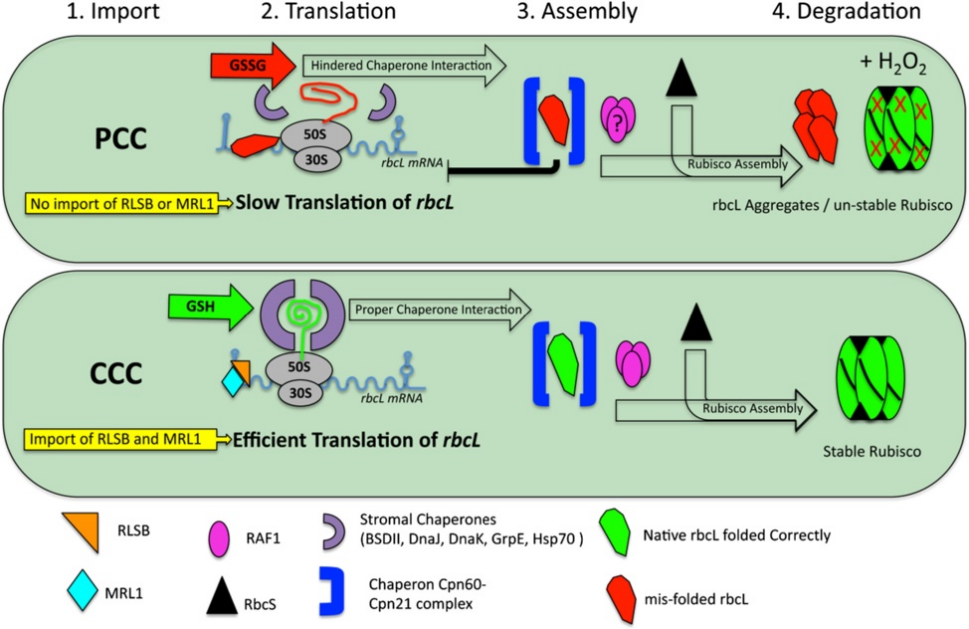
**Regulatory hypotheses for how Rubisco becomes selectively localized to the central compartment chloroplasts (CCC) of *Bienertia *chlorenchyma cells.** Regulatory point one, (yellow boxes) is the selective targeting or import of *rbcL* mRNA binding proteins (RBP) such as RLSB, or other proteins necessary for Rubisco assembly. Regulatory point two, is the translation of *rbcL,* where in the CCC under reduced conditions (GSH, green box) *rbcL* RBPs would increase translation of *rbcL*, while in the peripheral compartment chloroplasts (PCC) under elevated oxidized glutathione conditions (GSSG, red box), *rbcL* RBPs would not bind to *rbcL*, due to incorrectly folded rbcL binding to *rbcL* slowing translation. Regulatory point three, is the interaction of rbcL with stromal chaperonins and assembly of Rubisco, which in the PCC could be impeded due to a lack of chaperonins like RAF1 or the redox state of the chloroplast. Regulatory point four, is the stability or degradation of Rubisco which might be increased in the PCC due to elevated H_2_O_2_ concentrations. Figure adapted from [23,27,66].

In this study, mechanisms responsible for selective Rubisco accumulation in forming dimorphic chloroplasts of mature *Bienertia* cells were investigated, a process which is necessary to support C_4_ biochemistry. Development of *Bienertia* leaves occurs acropetally. During development of the two cytoplasmic domains in young *Bienertia* chlorenchyma, some Rubisco is initially present in PCC as well as CCC, while mature chlorenchyma cells have strong selective accumulation of Rubisco only in the CCC [22]. These observations coincide with younger chlorenchyma having *rbcL* transcripts in both chloroplasts, while in mature chlorenchyma the transcripts are selectively expressed in the CCC (N. Koteyeva and E. Voznesenskaya, unpublished results). Findings from this study support a model in which the posttranscriptional regulatory activity of RLSB, together with variations in the plastid redox state, could function synergistically to activate/stabilize *rbc*L mRNA along with Rubisco in the CCC. The opposite effects would occur in the PCC, where repression/degradation of *rbc*L mRNA and Rubisco destabilization would prevent its accumulation (see details in the model in Figure 6). In *Bienertia*, control of *rbc*L translation may occur in coordination with increased transcript stability, possibly mediated by RLSB, as proposed for *Zea mays*[26]. In this case during *Bienertia* cell maturation, RLSB abundance could become progressively more chloroplast specific, so that *rbc*L mRNA and its encoded protein both become more abundant in the CCC, and less abundant in the PCC, in coordination with changes in photosynthetic electron transport and redox status. Under altered redox conditions in the PCC, rbcL may bind to *rbcL*, and prevent the binding of stablizing RBPs (like MRL1 and RLSB) leading to decreased *rbcL* transcripts. Additionally, elevated H_2_O_2_ concentrations in the PCC, could interfere with Rubisco assembly or cause degradation leading to less Rubisco.

## Conclusions

The evidence presented here provides background that allows us to construct an initial model of selective Rubisco compartmentalization within single chlorenchyma cells of the single-cell C_4_ plant *Bienertia* (Figure 6). This model accounts for the fact that differential transcription of nuclear-encoded genes cannot be responsible for the selective compartmentalization of photosynthetic or regulatory proteins within a single cell. This model proposes that the *rbcL* post-transcriptional regulator factor RLSB (and possibly others), and differential redox status of the dimorphic chloroplasts, work together to restrict Rubisco accumulation to only one of the two chloroplast types of *Bienertia* chlorenchyma cells. This model is in concurrence with findings from many studies [27,70,71] demonstrating that selective accumulation of Rubisco in C_4_ systems is accomplished primarily by post-transcriptional mechanisms. Control of gene expression at this level is an absolute requirement to achieve the differential Rubisco accumulation patterns that characterizes C_4_ photosynthesis in *Bienertia.*

## Methods

### Plant material

*Bienertia sinuspersici* (hereafter referred to as *Bienertia*) plants were grown in controlled environmental chambers (Econair GC-16; Bio Chambers). Seedlings or vegetative cuttings were started under low light [100 PPFD (μmol quanta m^-2^ s^-1^) and temperature conditions with a day/night temperature of 25/22˚C and a photoperiod of 14/10 h]. Plants were moved to high light and temperature conditions (1000 PPFD, with a day/night temperature of 35/25˚C and a photoperiod of 14/10 h) once several branches were present. Mature leaves (~ 3 cm long) from 2- to 6- month old plants were routinely used for biolistic and protoplast transformation.

### Construct assembly

Fusion protein constructs were made by subcloning DNA fragments of interest into the 35S:puc18-spGFP6 vector using restriction enzymes *BamHI*/*NheI* for N-terminal GFP addition, and restriction enzymes *XbaI*/*SpeI* for the C-terminal addition of the *RbcS* 3′ un-translated region (UTR) (M. Tegeder, unpublished data) (New England BioLabs, Ipswich, MA). DNA fragments of *RbcS*, and *BADH* were obtained from a cDNA library prepared using CloneTech SMARTer PCR cDNA synthesis kit according to manufacture protocols (Mountain View, CA). The *PPDK* fragments were amplified from a previous c-DNA library preparation [29]. DNA fragments were amplified using gene specific primers with restriction enzyme cut sites added for CDS fragments, or with the smart oligo primer, with one gene specific primer (Additional file 1: Table S2). The PCR fragments were digested and ligated into the puc18 vector using T4 DNA ligase (New England BioLabs, Ipswich, MA). Plasmid DNA of all constructs was purified using Zymo Research Plasmid DNA miniprep kits (Irvine, CA) and verified by sequencing (WSU Sequencing Core) before transformation (sequence of DNA fragments used in constructs are shown in Additional file 7: Figure S6). The CDS for roGFP2 was inserted into the puc18 vector, by PCR amplifying the roGFP2 fragment from the pRSETB vector [31], using primers that added the restriction enzyme cut sites NheI and XbaI to the PCR fragment (Additional file 1: Table S2). The PCR fragment and puc18 vector were digested with NheI and XbaI, ligated together using T4 DNA ligase, and verified by sequencing. The addition of the plant super ubiquitin promoter (pSU) and pSU intron, to the full-length *RbcS*-GFP fragment was done by first cutting out the full-length *RbcS*-GFP fragment using the enzymes *BamHI* and *HindIII* and ligated into Tegeder vector 656 (M. Tegeder, unpublished data). The pSU promoter and intron were subsequently added to vector 656 using the enzymes SacII and BamHI from Tegeder vector 655 (M. Tegeder, unpublished data). The construct with the transit peptide of AGPase fused to GFP was from [28], to test targeting of the large subunit of AGPase.

### Biolistic transformation

Constructs were introduced into onion epidermal cells, spinach leaves, and *Bienertia* chlorenchyma cells by particle bombardment according to [72] and the manufacture’s protocol (Bio-Rad). For *Bienertia* bombardment, the epidermis of the leaf was first gently removed using forceps to grab the tip of the leaf and a small plastic pestle to gently press out chlorenchyma and internal leaf cells, resulting in an epidermis-less leaf. Cells were bombarded with 1 μm plasmid coated gold micro-carriers, using 1100 p.s.i. rupture discs at a distance of 6 cm from the stop screen. After bombardment epidermal free leaves were incubated overnight in a buffer (MES-NaOH, pH 5.8) that was osmotically adjusted with glycine betaine to match the osmolality of the leaf (determined with a 5500 Vapor Pressure Osmometer, Wescor). Bombarded onion epidermal cells, and spinach leaves were incubated overnight at room temperature on water moistened filter paper.

### Protoplast isolation

Intact protoplasts were obtained from mechanically isolated chlorenchyma cells, using the protocol similar to [21] and [40]. Briefly, the leaf osmolality of plant material was measured prior to isolation and subsequently all buffers were osmotically adjusted to the measured value. Approximately 150 mature leaves (longer than 1 cm) were removed using a razor blade, early in the photoperiod. Chlorenchyma cells were gently pressed out using a motor and pestle into 8 ml of protoplast buffer (PB) (5 mM MES-NaOH, pH 5.8, 10 mM CaCl_2_, 0.7 M Sucrose, 1% Dextran, and matching glycine betaine concentration). The isolated cells were filtered through a nylon mesh with 1-mm pore size to remove large leaf particles. Cells were washed twice with PB by removing cells from the top of the 15 mL tube after centrifugation (5 min, 3.5 g in a Damon IEC HN-SII centrifuge). 1 mL of cells was mixed with 1 mL of digestion buffer (PB plus 2% [w/v] Sumizyme C [Shin-Nihon Chemical], 0.25% [w/v] Macerase [Calbiochem], and matching glycine betaine concentration) in a 2 mL round-bottom centrifuge tubes. Cells were incubated for 40 minutes at 35˚C on a Gyromax 737 orbital incubator shaker (Amerex Instruments) at 65 rpm with illumination from a 100-W light bulb to obtain protoplasts. The enzyme/cell mixture was centrifuged at 100 g for 2 min in a benchtop centrifuge and the floating layer of protoplasts were collected and washed twice with PB.

### Protoplast transformation

The initial procedure used was developed based on [73], and subsequently modified using the protocol of [40]. Briefly, isolated protoplasts were incubated on ice for 30 min in W5 solution (5 mM MES-NaOH, pH 5.8, 154 mM NaCl, 125 mM CaCl_2_ and 5 mM KCl). Approximately 1.5 × 10^4^ protoplasts were resuspended in chilled-protoplast buffer (5 mM MES-NaOH pH 5.8, and matching glycine betaine concentration) and mixed with 5 μg of plasmid DNA. The protoplast/DNA mixture was gently mixed with PEG (Sigma-Aldrich, St. Louis, MO, USA) solution (40% [w/v] in PB) to initiate a 5 min transfection at room temperature. Transfected protoplasts were mixed with 5 volumes of protoplast buffer, centrifuged for 1 min at 15 g, resuspended in 100 μl of protoplast buffer and subsequently cultured overnight at 25°C with a light intensity of 25 μmol m^-2^ s^-1^.

### Protein immunolocalization in situ

Tissue was prepared and analyzed according to the procedures of [35]. Leaf samples for *Bienertia* and *S. eltonica* were fixed in FAA (50% ethanol, 5% glacial acetic acid, 10% formalin) fixative overnight at room temperature. Samples were dehydrated using increasing concentrations of ethanol, washed using CitriSolv, and embedded in Paraplast Plus. The paraffin-embedded samples were sectioned (thickness approximately 7.5 μm) using a rotary microtome, mounted on a poly-L-lysine-coated slides (Electron Microscopy Sciences, PA), and dried overnight at room temperature. After deparaffinization by CitriSolv, the sections were rehydrated through an ethanol series, and rinsed with H_2_O twice. Slides were washed twice with PBS buffer, and subsequently incubated with goat serum for 30 min at room temperature. Slides were washed twice with PBS buffer containing 0.1% BSA. Samples were incubated with diluted antibody for either, 2 h for PEPC and rbcL, or overnight for RLSB, in PBS buffer containing 0.1% BSA (PEPC was diluted 1:500; source [6], rbcL was diluted 1:1000; source [74], RLSB was diluted 1:250; source [26]. Slides were washed three times with PBS buffer containing 0.1% BSA, and incubated for 1 hour with the Alexafluor 546 conjugated secondary antibody (Invitrogen, Grand Island, NY), diluted 1:400 in PBS buffer containing 0.1% BSA. Samples were washed three times in PBS buffer containing 0.1% BSA, and subsequently rinsed three times in PBS buffer. Slides were mounted with vectashield (Vector Laboratories, CA), and sealed with a cover slip and nail polish, prior to visualization on the Ziess 710LSM confocal microscope (Imaging Facility, Dept. of Biological Sciences, University at Buffalo).

### Confocal microscopy

*Quantification of PSII Fluorescence* – This procedure was adapted from [33]. Microscopy of fresh protoplasts was carried out using a Laser-Scanning-Microscope LSM 510 invert (Carl Zeiss, Oberkochen, Germany). The excitation wavelength for chlorophyll autofluorescence was 544 nm from a HeNe laser and the emitted wavelength captured for the images was long-band pass filter at 654 nm (Carl Zeiss). Images were obtained by averaging 8 measurements in frame mode. In this mode, the frame is scanned repeatedly and the signal is averaged. To quantify the relative intensities of chlorophyll fluorescence on individual chloroplasts from the central and peripheral compartments, the lambda mode was used.

*GFP expression analysis* - Approximately 100 μl of protoplasts were imaged on cover glass slides using a Zeiss 510 LSM. Serial Z-stack images were acquired at 1 μm intervals using a 63× water-immersion lens at a digital resolution of 1024 × 1024. The excitation wavelength for chlorophyll autofluorescence was 544 nm and the emitted wavelength captured for the images was long-band pass filter at 654 nm. GFP was excited using a wavelength of 488 nm and the emission detected at a band path of 505**–**530 nm. All images were further processed and composed using Adobe Photoshop CS5 (Adobe Systems Incorporated, Seattle, USA). All experiments were repeated at least three independent times with similar results. For qualitative analysis of expression, lambda mode was used to assess the intensity of GFP fluorescence from each transformed *Bienertia* protoplast. Fluorescence intensity values were obtained using an excitation wavelength of 488 nm, and detection of emission at a wavelength of 513 nm. In each cell analyzed, the ratio of GFP fluorescence intensity (CCC/PCC) from measurements on individual chloroplasts was determined. The ratios were averaged across the number of quantified cells, and the results presented are from 7 biological replicates. Statistical analysis was performed using an independent t-test using the Stastica 7 software (StatSoft Inc., Tulsa, OK, USA).

### DAB staining

Mature leaves were cut into 3–4 mm sections using a super sharp razor blade. Leaf sections were placed into a glass vial for staining. Four stains (H_2_O_2_ control, H_2_O_2_ detection, Peroxidase control and Peroxidase detection) were performed using Sigma FAST 3, 3′ – Diaminobenzidine (DAB) Tablets (Sigma Aldrich, St. Louis MO) carried out at 25°C. Tablets were dissolved in water that contained a glycine betaine concentration that osmotically matched the leaves (DAB 0.7 mg ml^-1^, 7 mM H_2_O_2_, 60 mM Tris buffer pH = 7.5). For detection of the generation of H_2_O_2_ the tissue was incubated in the presence of DAB under 100 PPFD for 4 h (the control was without DAB). For peroxidase detection, leaf sections were incubated in the presence of DAB for 45 min with addition of 7 mM H_2_O_2_ under 10 PPFD (control was without H_2_O_2_). After staining leaves were fixed following [75]. After imbedding the plane of the cut leaf surface was sectioned down to using a Reichert Ultracut R ultramicrotome (Reichert-Jung GmbH, Heidelberg, Germany). The sample was super-glued (The Original Super Glue) onto an aluminum specimen mount (TED Pella) and imaged using the back-scattering detector under high vacuum mode at 30 kV accelerating voltage using a Quanta 200 F environmental field emission gun scanning electron microscope (FEI Company; Field Emission Instruments). Image quantification was done using Image J software on an equal number of PC and CC, typically 5–7 chloroplasts per cell depending on the number of PC present. A total of 3 cells were quantified per replicate, with two technical replicates, and two biological replicates being quantified. Statistical analysis was done using an independent t-test using the Stastica 7 software (StatSoft Inc., Tulsa, OK, USA).

## Abbreviations

AMP: Adenosine monophosphate; ATP: Adenosine-5'-triphosphate; BS: Bundle Sheath cell; CC: Central compartment; CCC: Central compartment chloroplast; CDS: Coding sequence; CES: Control by the epistasy of synthesis; DAB: 3,3-Diaminobenzidine; M: Mesophyll cell; NAD-ME: NAD-malic enzyme; PB: Protoplast buffer; PC: Peripheral compartment; PCC: Peripheral compartment chloroplast; PEP: phosphoenolpyruvate; PEPC: Phosphoenolypyruvate carboxylase; Pi: Orthophosphate; PPDK: Pyruvate,Pi dikinase; PPFD: Photosynthetic photon flux density, μmol quanta m^-2^ s^-1^; PPi: Pyrophosphate; PS I: Photosystem I; PS II: Photosystem II; rbcL: Rubisco large subunit mRNA; rbcL: Rubisco large subunit protein; RbcS: Rubisco small subunit mRNA; RbcS: Rubisco small subunit protein; RLSB: *rbcL* RNA S1-binding domain protein; Rubisco: Ribulose bisphosphate carboxylase-oxygenase; TRX: Thioredoxin.

## Competing interests

• In the past five years or in the future, no authors have received or will receive any reimbursements, fees, funding, or salary from an organization that may gain or lose financially from publication of this manuscript. No organization is financing this manuscript.

• No author holds any stocks or shares in an organization that, either now or in the future, may gain or lose financially from the publication of this manuscript.

• No author is currently applying for any patents relating to the content of the manuscript. No author has received reimbursements, fees, funding, or salary from an organization that holds or has applied for patents relating to the content of the manuscript.

• No author has any financial or non-financial competing interests to declare in relation to this manuscript.

## Authors’ contributions

JR carried out the assembly of constructs, biolistic and protoplast transformation, participated in the immunolocalization analysis and RLSB sequence analysis, performed the DAB histochemical analysis and drafted the manuscript. PY carried out the immunolocalization analysis, assisted in RLSB sequence analysis and editing of manuscript. JB participated in design and coordination, assisted with the immunolocalization analysis, and helped in writing the manuscript. TO participated in the design of the study. GE conceived the study, participated in the design and coordination, and helped draft the manuscript. All authors have read and approved the final manuscript.

## Supplementary Material

Additional file 1: Table S1 List of constructs and summary of results from GFP expression. **Table S2.** List of primer sequences used for this study. Click here for file

Additional file 2: Figure S1 Biolistic results with puc18 spGFP. Click here for file

Additional file 3: Figure S2 Biolistic results with PPDK-180 spGFP. Click here for file

Additional file 4: Figure S3 Biolistic results with RbcS-273 spGFP. Click here for file

Additional file 5: Figure S4 Biolistic results with RLSB spGFP. Click here for file

Additional file 6: Figure S5 Representative image for quantification of GFP fluorescence from RbcS-FL spGFP and RbcS-FL roGFP2. Click here for file

Additional file 7: Figure S6 DNA sequences used in constructs. Click here for file
