# Supplementary material for: Exploring mechanisms linked to differentiation and function of dimorphic chloroplasts in the single cell C4 species Bienertia sinuspersici
Source: BMC Plant Biol. 2014 Jan 21;14:34. doi: 10.1186/1471-2229-14-34 (PMC3904190; doi:10.1186/1471-2229-14-34)
Supplement: Additional file 1: Table S1 — List of constructs and summary of results from GFP expression. Table S2. List of primer sequences used for this study. [file 1471-2229-14-34-S1.docx]

Supplemental Table 1. Table showing constructs used to test protein-targeting hypothesis and the results from confocal analysis, using biolistic transformation of onion epidermal cells, *Bienertia* chlorenchyma, and protoplast transformation of *Bienertia*. For each construct it is indicated where import was observed. n.d. = no data, n.s. = not shown. The number after each enzyme name represent the number of nucleotides present (T.P. = Transit Peptide, CDS = Coding Sequence, F.L.= Full length (5'UTR, CDS, & 3' UTR), Puc18 = 35S promoter with TNOS termination sequence, pSU = Super Ubiquitin Promoter with intron present before coding sequence and a TNOS termination sequence). * Choi et al (2001).

| Construct Name | Biolistic Onion Epidermal Cells | Biolistic Bienertia Chlorenchyma | Protoplast Bienertia Chlorenchyma | Results shown |
| --- | --- | --- | --- | --- |
| Puc18 spGFP | No-Import | No-Import | No-Import | Figure 1 |
|  |  |  |  |  |
| BADH 180 spGFP | No-Import | No-Import | n.d. | n.s. |
| BADH 273 spGFP | No-Import | No-Import | n.d. | n.s. |
| BADH CDS spGFP | Import | No-Import | Import | n.s. |
|  |  |  |  |  |
| PPDK 180 spGFP | Import | Rare Import | Import | Figure 1 |
| PPDK 273 spGFP | Import | No-Import | Import | n.s. |
| PPDK CDS spGFP | Import | No-Import | n.d. | n.s. |
|  |  |  |  |  |
| RbcS 252 spGFP | No-Import | No-Import | n.d. | n.s. |
| RbcS 273 spGFP | Import | Rare-Import | Import | Figure 1 |
| RbcS CDS spGFP | Import | No-Import | Import | n.s. |
|  |  |  |  |  |
| 5' UTR RbcS 273 spGFP | Import | No-Import | Import | n.s. |
| 5' UTR RbcS CDS spGFP | Import | No-Import | Import | n.s. |
| RbcS 273 3' UTR spGFP | Import | No-Import | Import | n.s. |
| RbcS CDS 3' UTR spGFP | Import | No-Import | Import | n.s. |
| RbcS F.L. spGFP | Import | No-Import | Import | Figure 2 |
| pSU RbcS F.L. spGFP | Import | n.d. | Import | Figure 1 |
| RbcS F.L. roGFP2 | Import | n.d. | Import | Figure 2 |
|  |  |  |  |  |
| RLSB CDS spGFP | Import | n.d. | n.d. | Supl. Fig. 5 |
|  |  |  |  |  |
| AGPase TP spGFP* | Import | No-Import | Import | n.s. |
|  |  |  |  |  |

Supplemental Table 2. Table showing primers used in the generation of GFP-fusion constructs used to test protein-targeting hypothesis. The number after each enzyme name represents the number of nucleotides present (CDS = Coding Sequence, F.L.= Full length (5'UTR, CDS, & 3' UTR)). Primer nucleotides in bold represent DNA sequence not present in the gene, and bold italicized base pairs show restriction enzyme cut sites.

| **Primer Name** | **Primer Sequence 5' --> 3'** | **Constructs made using this primer** |
| --- | --- | --- |
| PPDK FW | **GATC*GGATCC***ATGGCATTATGTTTCAAAGG | PPDK 180 spGFP, PPDK 273 spGFP, PPDK CDS spGFP |
| PPDK 180 RV | **GATC*GCTAGC***GTTGGACTGGCTCTGGCTAG | PPDK 180 spGFP |
| PPDK 273 RV | **GATC*GCTAGC***GTCCCCGTCACTTCTTCCTTT | PPDK 273 spGFP |
| PPDK CDS RV | **GATC*GCTAGC***AACCGCAACTTGAGCTGC | PPDK CDS spGFP |
|  |  |  |
| SSU FW | **GATC*GGATCC***ATGGCTTCCAGTTTGAT | RbcS 252 spGFP, RbcS 273 spGFP, RbcS CDS spGFP |
| SSU 252 RV | **GATC*GCTAGC***GGATTCTGTAGATAGAGGTG | RbcS 252 spGFP |
| SSU 273 RV | **GATC*GCTAGC***GTATTGGATCTCACGCAACAA | RbcS 273 spGFP, 5' UTR RbcS 273 spGFP |
| SSU CDS RV | **GATC*GCTAGC***GTAGCCTGGGGGCTTGTAGG | RbcS CDS spGFP, 5' UTR RbcS CDS spGFP |
| SSU 3'UTR FW | **GATC*TCTAGA***TCAATGTATCAATTTATATA | RbcS CDS 3' UTR spGFP |
|  |  |  |
| P44 FW | **GATC*GGATCC***AATGCCCGTTGTGATGAAATCT | RLSB CDS spGFP |
| P44 1954 RV | **GATC*GCTAGC***ATGAACATTTTAAGATGCCC | RLSB CDS spGFP |
|  |  |  |
| roGFP2 FW | **GATC*GCTAGC***ATGAGTAAAGGAGAAGAACT | RbcS F.L. roGFP2 |
| roGFP2 RV | **GATC*TCTAGA***TTATTATTTGTATAGTTCAT | RbcS F.L. roGFP2 |
|  |  |  |
| Smart Oligo FW | **GATC*GGATCC***ATCAACGCAGAGTACGCGGG | 5' UTR RbcS 273 spGFP, 5' UTR RbcS CDS spGFP, RbcS F.L. spGFP, RbcS F.L. roGFP2 |
| Smart Oligo RV | **GATC*GTCGAC***GGTATCAACGCAGAGTACT | RbcS 273 3' UTR spGFP, RbcS CDS 3' UTR spGFP, RbcS F.L. spGFP, RbcS F.L. roGFP2 |
|  |  |  |
| BADH FW | **GATC*GGATCC***ATGTCGATCCCTATACCTTC | BADH 180 spGFP, BADH 273 spGFP, BADH CDS spGFP |
| BADH 180 RV | **GATC*GCTAGC***CCTCTTCAGTGCTCTTCGAG | BADH 180 spGFP |
| BADH 273 RV | **GATC*GCTAGC***ATGGTCTTTTTTTTCTGATAC | BADH 273 spGFP |
| BADH CDS RV | **GATC*GCTAGC***AGGAGAATTGTACCATCC | BADH CDS spGFP |
|  |  |  |
