# Supplementary material for: Exploring mechanisms linked to differentiation and function of dimorphic chloroplasts in the single cell C4 species Bienertia sinuspersici
Source: BMC Plant Biol. 2014 Jan 21;14:34. doi: 10.1186/1471-2229-14-34 (PMC3904190; doi:10.1186/1471-2229-14-34)

Supplemental Figure 1. Biolistic transient expression analysis of the spGFP construct (positive control, no transit peptide sequence), showing no plastid import in an onion epidermal cell (A-C), a spinach mesophyll cell (D–F), and a *Bienertia* chlorenchyma cell (G–I). Images A, D, & G are emission of GFP. Images B, E, & H are emission of chlorophyll auto-fluorescence. Images C, F, & I are merged images of GFP expression and chlorophyll auto-fluorescence. CCC = central compartment chloroplast, PCC = peripheral compartment chloroplast. Scale Bar = 50 µm.


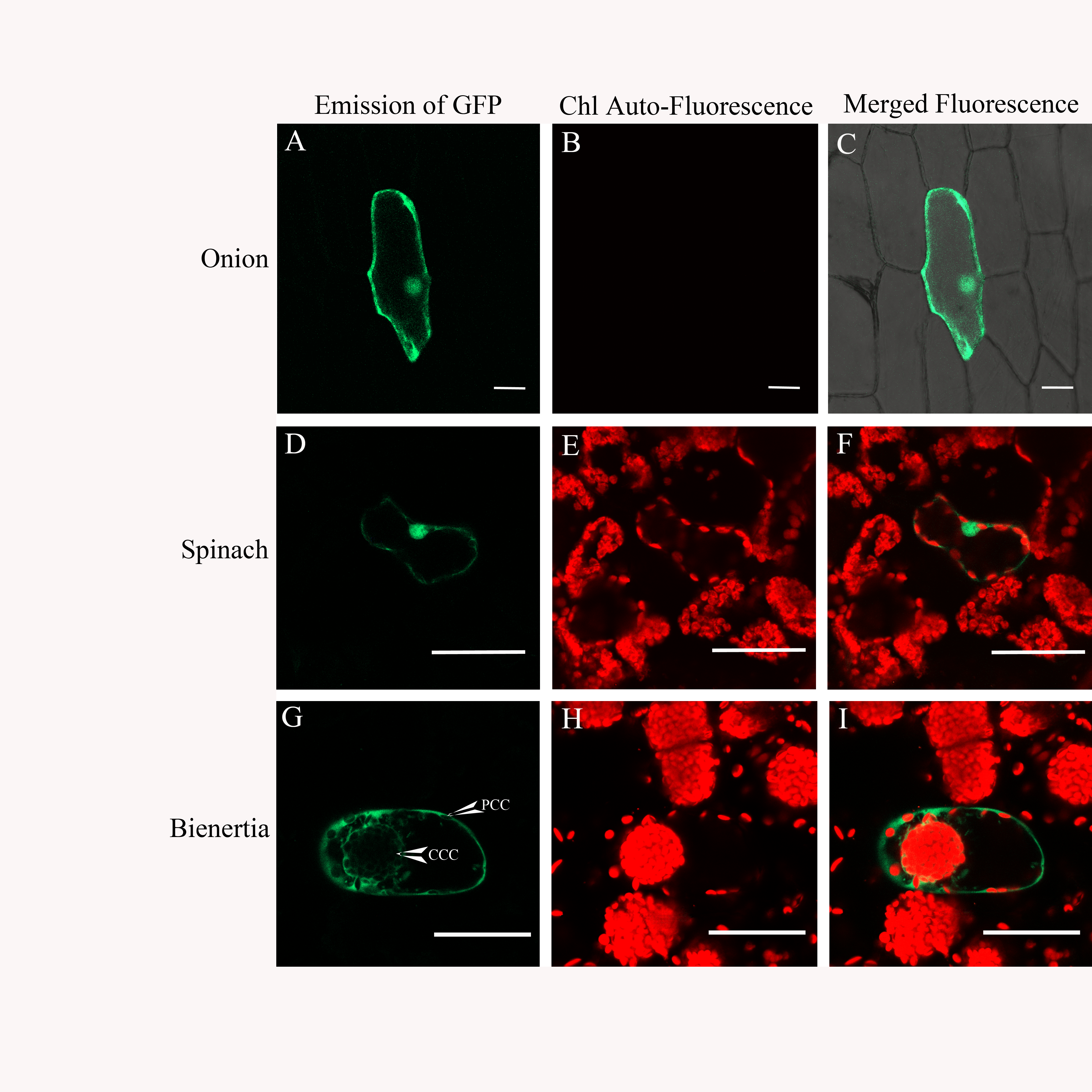

Supplement: Additional file 2: Figure S1 — Biolistic results with puc18 spGFP. [file 1471-2229-14-34-S2.docx]
