# Supplementary material for: Exploring mechanisms linked to differentiation and function of dimorphic chloroplasts in the single cell C4 species Bienertia sinuspersici
Source: BMC Plant Biol. 2014 Jan 21;14:34. doi: 10.1186/1471-2229-14-34 (PMC3904190; doi:10.1186/1471-2229-14-34)

Supplemental Figure 3. Biolistic expression of the SSU 273–spGFP construct showing plastid import in an onion epidermal cell (A-C), chloroplast import in a spinach mesophyll cell (D–F), and *Bienertia* where in most transformed chlorenchyma cells chloroplast import was not observed as illustrated here (G-I), but occasionally chloroplast import into both chloroplasts was observed (J–L). Images A, D, G, & J are emission of GFP. Images B, E, H, & K are emission of chlorophyll autofluorescence. Images C, F, I, & L are the merged images of GFP expression and chlorophyll autofluorescence. CCC = central compartment chloroplast, PCC = peripheral compartment chloroplast. Scale Bar = 50 µm.


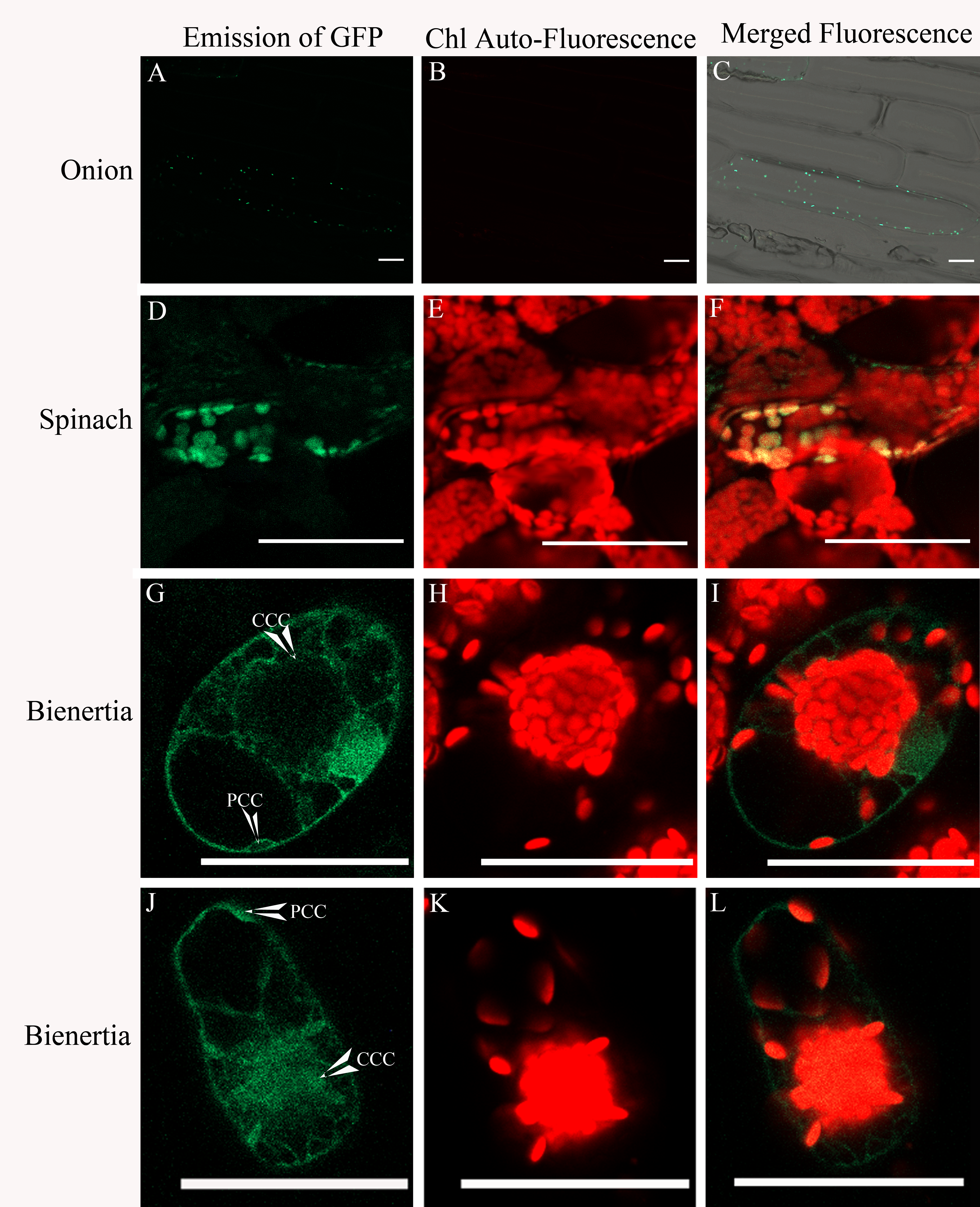

Supplement: Additional file 4: Figure S3 — Biolistic results with RbcS-273 spGFP. [file 1471-2229-14-34-S4.docx]
