# Supplementary material for: Exploring mechanisms linked to differentiation and function of dimorphic chloroplasts in the single cell C4 species Bienertia sinuspersici
Source: BMC Plant Biol. 2014 Jan 21;14:34. doi: 10.1186/1471-2229-14-34 (PMC3904190; doi:10.1186/1471-2229-14-34)

Supplemental Figure 4. Biolistic expression of the RLSB–spGFP construct showing plastid import in onion epidermal cell (A&B). Image A is emission of GFP. Image B is the merged image of GFP expression and the bright-field view. Scale Bar = 100 µm.


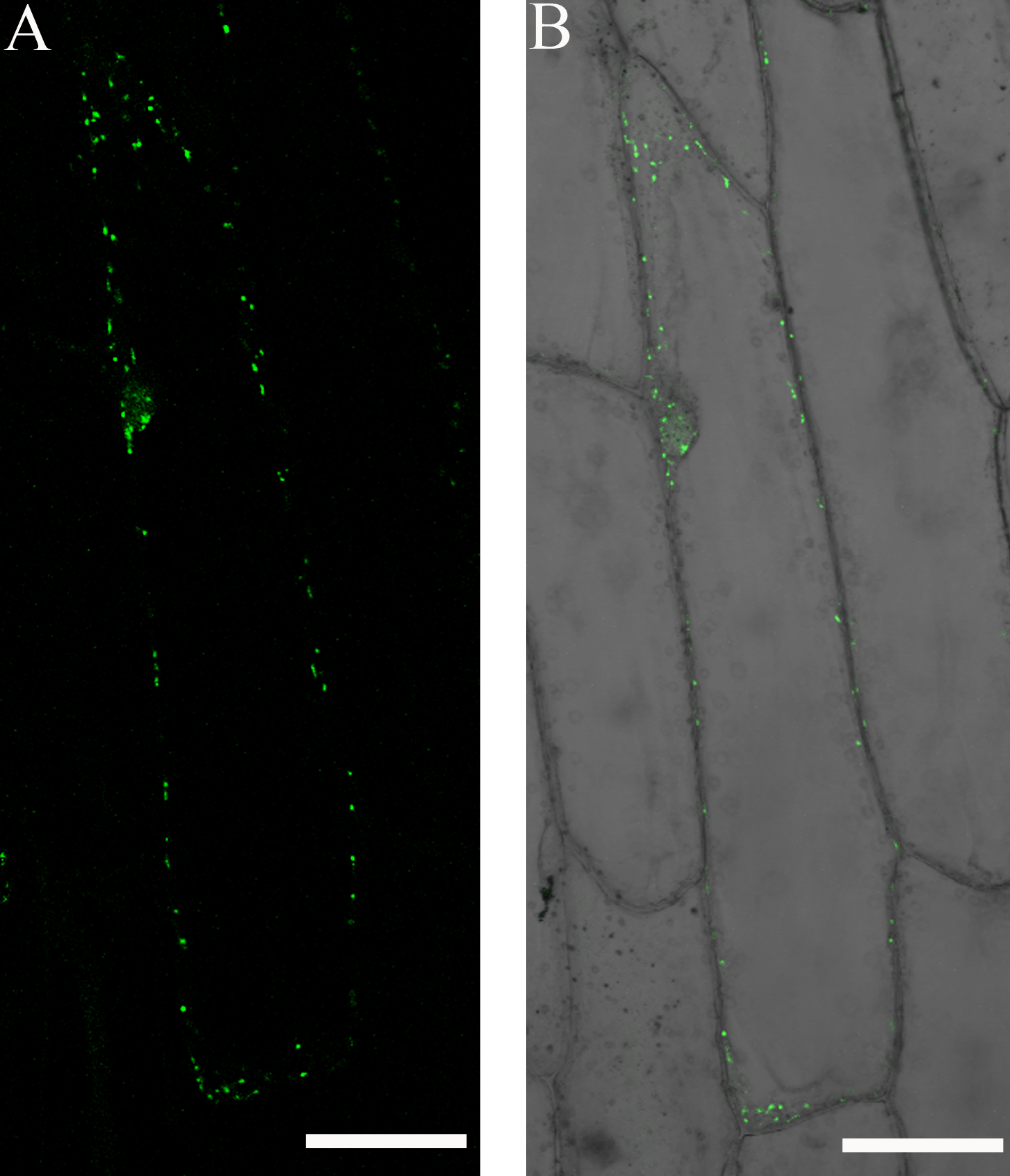

Supplement: Additional file 5: Figure S4 — Biolistic results with RLSB spGFP. [file 1471-2229-14-34-S5.docx]
