# Supplementary material for: Exploring mechanisms linked to differentiation and function of dimorphic chloroplasts in the single cell C4 species Bienertia sinuspersici
Source: BMC Plant Biol. 2014 Jan 21;14:34. doi: 10.1186/1471-2229-14-34 (PMC3904190; doi:10.1186/1471-2229-14-34)

Supplemental Figure 5. Confocal microscopy quantification of GFP fluorescence from *Bienertia* protoplasts expressing the RbcS-FL spGFP (A & B) or RbcS-FL roGFP2 (C & D). The roGFP2 protein is redox sensitive while the spGFP protein is redox insensitive. Protoplasts were scanned for a focal plane closest to the slide surface to maximize GFP fluorescence from each chloroplast before quantification. Cursors’ were placed on captured image to maximize GFP fluorescence intensity in their respective compartment. Each image had four points of analysis; one central compartment chloroplast, one peripheral chloroplast, one spot inside the cell cytosol (cell background), and one spot outside the cell (media background). Results from qualitative analysis of multiple cells are shown in Table 1. Image A shows GFP expression from RbcS-FL spGFP construct; cursor 1 is on peripheral chloroplast, cursor 2 is on a central compartment chloroplast, cursor 3 is the cell background, and cursor 4 is the media background. Panel B has labeled traces showing the intensity of fluorescence from each cursor in Image A. Image C shows GFP expression from the RbcS-FL roGFP2 construct; cursor 1 is on a central compartment chloroplast, cursor 2 is on a peripheral chloroplast, cursor 3 is the cell background, and cursor 4 is the media background. Panel D has labeled traces showing the intensity of fluorescence from cursors in Image C.


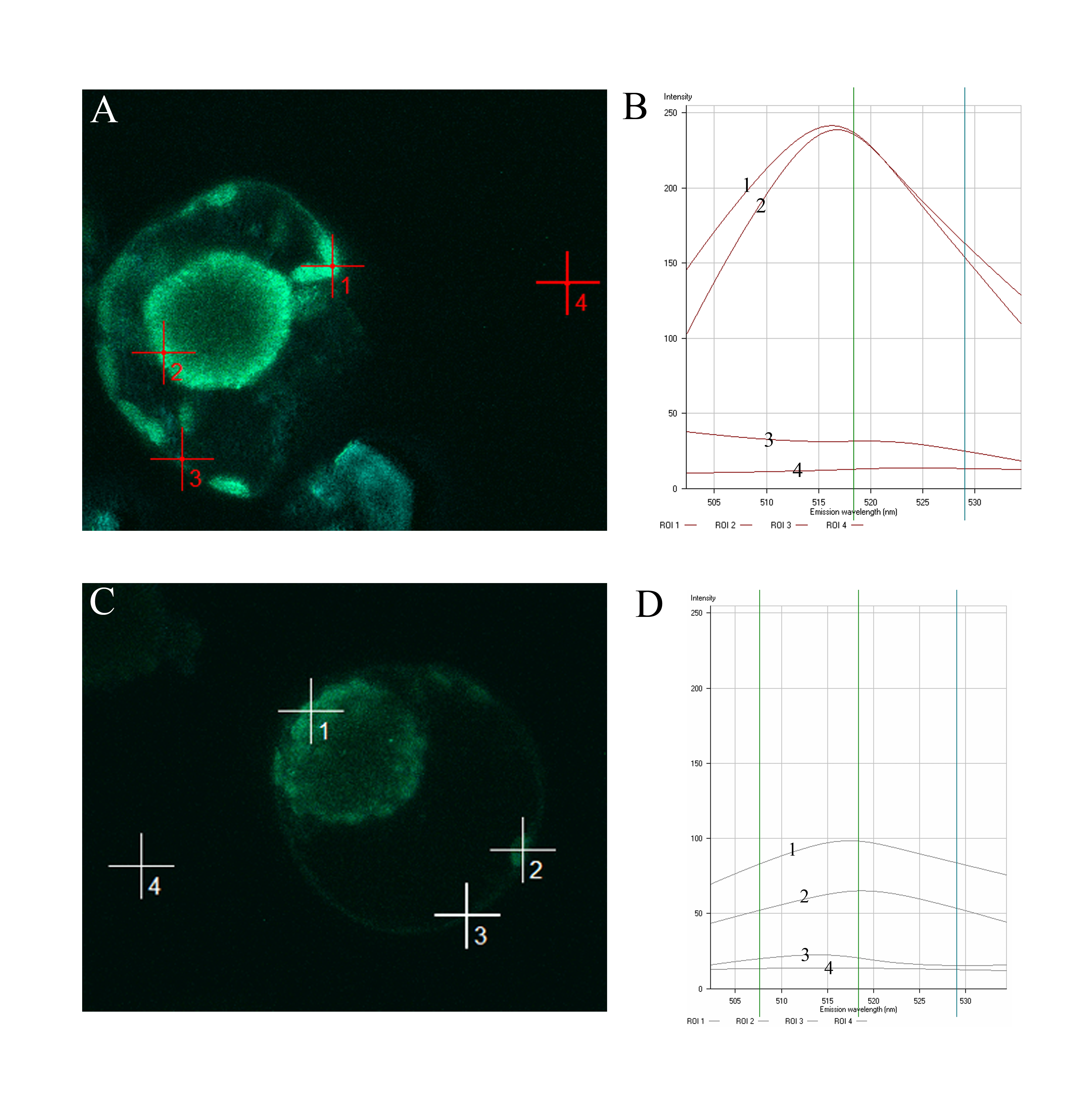

Supplement: Additional file 6: Figure S5 — Representative image for quantification of GFP fluorescence from RbcS-FL spGFP and RbcS-FL roGFP2. [file 1471-2229-14-34-S6.docx]
