# Supplementary material for: Exploring mechanisms linked to differentiation and function of dimorphic chloroplasts in the single cell C4 species Bienertia sinuspersici
Source: BMC Plant Biol. 2014 Jan 21;14:34. doi: 10.1186/1471-2229-14-34 (PMC3904190; doi:10.1186/1471-2229-14-34)
Supplement: Additional file 7: Figure S6 — DNA sequences used in constructs. [file 1471-2229-14-34-S7.docx]

Supplemental Figure 6. Sequence of *Bienertia* DNA fragments used to generate GFP fusion constructs. A) DNA sequence for *RbcS*, B) DNA sequence for PPDK, C) DNA sequence for BADH. The un-translated regions (UTRs) of *RbcS* are italicized, the start codons are in green bold font, and the stop codons are in bold red font.

A)

*TCAGAGATCAAACTTACCAAGAATCATAGAGAGTGAGAGGTAAAC***ATG**GCTTCTAGTTTGATGTCCAGCGCCGCCACTGCCGCCGTCGCTACCGCTGCTAGTGCTCAGGCAGACATGGTGGCTCCATTCAATGGGTTGAAGTCCACTTCAGCTTTCCCTGTTACCAGGAAAAGCAACAATGACATCACTTCTCTTCCTAGCAATGGTGGAAAAGTCCAATGCATGCAGGTATGGCCACCAATTGGCAAGAAGAAGTTCGAGACCCTTTCTTACCTTCCACCCCTGTCTACTGAATCCTTGTTGCGTGAGATCCAATACCTTCTTAACAAGGGTTGGGTACCTTGCTTAGAATTTGAGCCAGAACACGGATTTGTGTATCGTGAGAACCACAGGTCACCAGGGTACTACGACGGACGTTACTGGACCATGTGGAAGCTCCCCATGTTCGGATGCACCGACCCAGCTCAGGTTGTGAACGAGCTCGAGGAGGCCAAGAAGGCTTACCCAAAAGCCTTCATCCGGATCATTGGATTCGACAACGTGCGCCAAGTCCAGTGCATTAGTTTCATTGCCTACAAGCCACCAGGCTAC**TAA***TCAATGTATCAATTTATATATGTATGCCTGGCTTAGTTTGTGGTTTATTTATTTCAATTCGGTCCAACCGTTTATGTAAGCATAATGACCAATCTTTCATTTCCAATTGCTTGTTGTTTCGCCTATGAAATTCGTGTTGGTTTTA*

B)

ATGGCATTATGTTTCAAAGGAATGCTAATCAGATCTGCTCCAGATGTATTTACACATACACTTGGTTATATGAAGGACCAATATCAAGTTGGTTGTAGCCAATGCAATAGTTTTCAGCGTGTTCAATTCCGGAACAGGAGGAGATGCCCACATCGATTAACTAGCCAGAGCCAGTCCAACAGACAAGATGTCATGGCTTTGATCTCAGACCCAGTTTCAACCACCACACAGCAGCGAGTATTTACCTTTGGTAAAGGAAGAAGTGACGGGGACAAGAGCATGAAGTCCTTGTTGGGAGGTAAAGGAGCAAATCTTGCAGAAATGGCAAGCATAGGTTTATCTGTTCCTCCTGGGTTGACCATTTCTACAGAAGCATGCCAAGAGTATCAGGACAGTGGCAAAATGCTTCCTGAAAGTCTGTGGGAGGAAATCCTTGAGGGCTTGAGGGTTATAGAGAGTGACATGGGAGCATACCTTGGAGACTCCTCTACACCTCTTCTACTTTCAGTTCGTTCTGGTGCTGCGATTTCTATGCCAGGGATGATGGACACTATCCTTAATCTTGGACTTAATGATGAAGTAGTTTCAGGGCTCGCCGCAAAGAGTGGAGAACGCTTTGCCTATGATTCATTTAGGCGCTTCTTGGACATGTTTGGTTGTGTAGTCATGGGTATTCCTCACTCATCATTCGAAGAGAAGTTAGAAAAGCTGAAGCAAATGAAAGGAGTTAAACTTGATACTGAGCTAACGGCATCTGATCTGAAAGAACTTGCAGAGCAGTACAAGAATGTGTATATTGAAACAAAAGGAGAGGTTTTTCCTGCTGATCCAAAGAAACAGCTTCAGTTAGCTGTTCAAGCAGTTTTTGATTCCTGGGACAGTCCAAGAGCTGTTAAGTATCGGAGCATCAACCAGATTTCTGGTTTGAAAGGCACTGCAGTTAATATTCAGTCTATGGTGTTTGGAAATATGGGGAATACTTCAGGAACAGGTGTTCTTTTTACTCGAAATCCAAACACTGGAGAAAGGAAACTCTATGGAGAGTTTTTAATTAATGCTCAGGGAGAAGACGTAGTCGCTGGCATTAGAACACCAGAAGACTTGGACACCATGAAGAGCTGCATGCCTGAAGCTTACACTGAACTTGTGCAAAACTGTGAAATTTTAGAGCAACATTATAAGGATATGATGGATATTGAATTCACTGTTCAAGAAAATAGGCTGTGGATGTTGCAATGCCGCTCTGGTAAGCGAACTGGAAAAGGTGCAGTGAAGATAGCTATAGATATGGTGAATGAAGGAAAAATCAACTCTCGGACTGCCATTAAGATGGTGGAACCACAACATCTTGATCAGCTTCTTCACCCACAGTTTGAAGACGCGTCAGCATATAAAGAAAGAGTGATTACCAGTGGACTGCCAGCATCTCCTGGAGCTGCTGTTGGGCAGATTGTATTCAGTGCTTATGATGCTGAAGCGTGGCATGCACAAGGGAAGAGTGCCATTCTGGTAAGGAATGAAACTAGCCCAGAGGATGTAGGGGGCATGCATGCGGCTGCTGGAATCTTGACCGCTAGGGGTGGAATGACGTCTCATGCTGCAGTTGTGGCTCGTGGCTGGGGAAAGTGTTGTGTTTCAGGGTGCTCTGAGATCAAAGTAAATGAAACTAACAAGAGTCTTGTGGTTGGAAACAATGTGCTAACTGAAGGGGATTGGCTTTCTCTTAATGGATCTACTGGTGAAGTGATCTTAGGGAAAGAACCGCTTTCACCACCTGCACTTAGTGGAGATTTGGAAACTATCATGTCTTGGACTGATGCTGTACGACGCCTTAAGGTTATGGCCAATGCAGATACACCAGAAGATGCTTTGGCAGCAAGAAACAATGGTGCTGAAGGGATTGGACTTTGTAGGACTGAGCACATGTTTTTTGCTTCAGATGACAGGATAAAAGCAGTGAGAAAAATGATAATGGCAGTTACACCTGAGCAGAGGAAAGCAGCTTTGGATCAGCTCTTACCTTACCAGAGATCTGACTTTGAGGGGATTTTTCGAGCAATGGATGGGCTTCCTGTGACAATTAGATTGCTAGACCCTCCGTTGCATGAGTTTCTTCCAGAAGGTGACTTGCAACAAATTGTGAATGAGCTAGCCTCTGAAACTGGCATTACAGAAGATGAAGTCTATTCCAGGGTAGAAAAGTTGTCAGAAGTTAATCCCATGTTGGGTTTCAGGGGATGCAGGTTGGGGATATCATATCCAGAACTAACGGAAATGCAAGCACGTGCTGTATTCCAAGCTGCATTGGGGATATCATATCCAGAACTAACGGAAATGCAAGCACGTGCTGTATTCCAAGCTGCAGTTTCAATGACCAACCAAGGCATTACAGTTCTACCGGAGATTATGGTTCCTCTTGTTGGAACACCTCAGGAGTTAGGACATCAAGTGAATTTGATACGAAGCGTTGCAACGAAGGTATTCTCGGAGATGGGTTCCTCAGTACGTTACAAAGTAGGAACAATGATTGAGATCCCTAGAGCAGCTCTAGTTGCGGACGAGATTGCAGTGGAAGCGGATTTCTTCTCTTTTGGAACGAATGATCTCACACAGATGACATTCGGGTATAGTAGAGATGATGTTGGCAAGTTTCTCCCTATATACTTGTCTAACGGCATACTGCAAACTGACCCCTTTGAGGTTCTGGACCAAAAAGGTGTGGGCCAACTCATCAAACTTGCAACCGAGAAAGGGCGTGCAGCAAAGCCTAGCTTAAAGGTGGGCATATGCGGAGAGCATGGAGGAGAGCCTTCTTCTGTTGCATTTTTTGCAGAGGCTGGACTTGATTACGTTTCATGTTCCCCATTTAGAGTTCCCATTGCAAGACTAGCAGCAGCTCAAGTTGCGGTT**TGA**

C)

**ATG**TCGATCCCTATACCTTCTCGTCAACTATTCATTGATGGAGAATGGAAAGAACCCATTAAAAGAAATCGTATCCCCATTATTAATCCTTCCACTGAAGAGACTATTGGTGAAATTCCAGCAGCTACTGCTGAAGATGTTGAGGCAGCAGTAAGTGCAGCTCGAAGAGCACTGAAGAGGAATAAGGGGAGAGATTGGGCTGCAACTTCTGGAGCTCATCGAGCAAGATACTTGCGTGCTATTGCTGCTAAGGTATCAGAAAAAAAAGACCATTTTGTAAAACTTGAAACCATGGATTCTGGGAAGCCACTGGATGAAGCAGTGTTGGACATAGATGATGTTTCGACATGTTTTGAATATTTTGCTGGTCAAGCAGAAGCTCTGGACAACAAGCAAAAGTATCCAGTCCAACTTCCTATGGATAGATTCAAAAGTCATGTTCTTAGGCAGCCTATTGGTGTTGTGGGTTTAATTTCCCCATGGAATTACCCACTTCTAATGGCTACATGGAAAATTGCTCCAGCTCTTGCTGCAGGATGTACAGCTGTACTTAAGCCATCTGAGTTGGCATCTGTGACTTGTCTAGAATTCGGTGAAGTTTGCAACGAAGTAGGACTTCCTCCTGGTGTGCTAAATATTTTGACAGGATTAGGTCCAGATGCTGGTGCCCCATTAGTGGCTCATCCTGATGTTGACAAGGTTGCATTTACTGGGAGTAGTGCTACTGGCAGCAAGGTTATGGGTTCTGCTGCCCAATTGGTAAAGCCTGTTACATTAGAACTCGGAGGTAAAAGTCCTATAATTGTGTTTGAAGATGTTGTTGACCTTGATGTAGCTGCTGAATGGACTATTTTTGGTTGTTTCTGGACAAATGGTCAAATATGCAGCGCAACATCTAGACTGCTTGTGCATGAAAGTATTGCAGCTGACTTTGTTGATAAGCTTGTAAAATGGACCAAGAAAATAAAGATTTCTGATCCATTTGAAGAAGGATGCCGGCTTGGCCCTGTTATTAGCAAAGGACAGTATGACAAAATTATGAAGTTTATATCGACAGCAAAGAGTGAAGGAGCAACTATCTTGTATGGAGGTTCCCGTCCTGAGCATTTGAAGAAAGGGTATTACATTGAGCCAACCATTGTAACTGATATCACCACATCCATGCAAATATGGAGGGAGGAAGTTTTTGGCCCCGTCTTATGTGTTAAAACATTTACTACTGAAGAAGAAGCCCTCGAATTGGCAAATGAAACAGAGTATGGTTTGGCTGCTGCTGTTTTTTCTAAAGACCCTGAAAGGTGTGAGAGGGTAACGAAGGCTCTAGAAGTTGGAGCTGTTTGGGTGAATTGCTCACAACCGTGCTTTTGCCATGCTCCATGGGGAGGCGTCAAGCGTAGTGGTTTTGGACGTGAGCTTGGAGAATGGGGTATCGAGAATTACTTGAATATCAAGCAGGTGACTAGCGACATTTCCGATGAACCTTGGGGATGGTACAATTCTCCT**TAA**
